# Adolescent and young adult glioma: systematic review of demographic, disease, and treatment influences on survival

**DOI:** 10.1093/noajnl/vdac168

**Published:** 2022-10-22

**Authors:** Armaan K Malhotra, Vishwathsen Karthikeyan, Veda Zabih, Alexander Landry, Julie Bennett, Ute Bartels, Paul C Nathan, Uri Tabori, Cynthia Hawkins, Sunit Das, Sumit Gupta

**Affiliations:** Division of Neurosurgery, University of Toronto, Toronto, Ontario, Canada; Division of Neurosurgery, University of Toronto, Toronto, Ontario, Canada; Division of Hematology/Oncology, The Hospital for Sick Children, Toronto, Ontario, Canada; Division of Neurosurgery, University of Toronto, Toronto, Ontario, Canada; Division of Hematology/Oncology, The Hospital for Sick Children, Toronto, Ontario, Canada; Division of Hematology/Oncology, The Hospital for Sick Children, Toronto, Ontario, Canada; Division of Hematology/Oncology, The Hospital for Sick Children, Toronto, Ontario, Canada; Division of Hematology/Oncology, The Hospital for Sick Children, Toronto, Ontario, Canada; Division of Paediatric Laboratory Medicine, The Hospital for Sick Children, Toronto, Ontario, Canada; Division of Neurosurgery, St. Michael’s Hospital, University of Toronto, Toronto, OntarioCanada; Division of Hematology/Oncology, The Hospital for Sick Children, Toronto, Ontario, Canada

**Keywords:** adolescents, glioma, prognostic factors, treatment, young adults

## Abstract

**Background:**

Prognostic factors in adolescent and young adult (AYA) glioma are not well understood. Though clinical and molecular differences between pediatric and adult glioma have been characterized, their application to AYA populations is less clear. There is a major need to develop more robust evidence-based practices for managing AYA glioma patients.

**Methods:**

A systematic review using PRISMA methodology was conducted using multiple databases with the objective of identifying demographic, clinical, molecular and treatment factors influencing AYA glioma outcomes.

**Results:**

40 Studies met inclusion criteria. Overall survival was highly variable across studies depending on glioma grade, anatomic compartment and cohort characteristics. Thirty-five studies suffered from high risk of bias in at least one domain. Several studies included older adults within their cohorts; few captured purely AYA groups. Despite study heterogeneity, identified favorable prognosticators included younger age, higher functional status at diagnosis, low-grade pathology, oligodendroglioma histology and increased extent of surgical resection. Though isocitrate dehydrogenase (IDH) mutant status was associated with favorable prognosis, validity of this finding within AYA was compromised though may studies including older adults. The prognostic influence of chemotherapy and radiotherapy on overall survival varied across studies with conflicting evidence.

**Conclusion:**

Existing literature is heterogenous, at high risk of bias, and rarely focused solely on AYA patients. Many included studies did not reflect updated pathological and molecular AYA glioma classification. The optimal role of chemotherapy, radiotherapy, and targeted agents cannot be determined from existing literature and should be the focus of future studies.

**Key Points**

- High-quality evidence on prognosticators in AYA glioma is lacking.
- Literature to date is heterogenous, rarely focused only on AYA, and prone to bias/confounding.
- Optimal role of chemotherapy and radiation cannot be determined.

**Importance of Study**

Glioma is a major contributor to oncologic morbidity and mortality in the adolescent and young adult (AYA) demographic. Historically, AYA have been poorly represented in glioma research due to limited enrollment and representation in both pediatric- and adult-focused cohorts. This systematic review synthesizes available prognostic, treatment and survival data for AYA glioma patients. We demonstrate the favorable impact of younger age and higher Karnofsky Performance Status (KPS) on overall survival (OS) and event-free survival (EFS). This review identified a positive association with OS and EFS with low-grade histology, oligodendroglial histology, isocitrate dehydrogenase (IDH) mutant molecular status and extent of surgical resection, though many included studies exhibited high bias risk and included older adults. It also highlights limited consensus on the role of adjuvant chemotherapy and radiotherapy in this population.

Gliomas represent a diverse histologic group of central nervous system tumors (CNS) with substantial molecular heterogeneity. Taken together, gliomas represent 29–35% of central nervous system tumors within the adolescent and young adult (AYA) demographic, of which two-thirds have been categorized as low-grade or World Health Organization (WHO) grade 1 or 2 with the remainder either WHO grade 3 or 4.^1^ Grade alone inadequately captures the biologic and molecular complexity of these cancers, particularly among low-grade gliomas (LGG).

Studies have demonstrated distinct clinical trajectories and underlying molecular influences in pediatric vs. adult LGG. While childhood LGG have limited propensity to undergo malignant transformation, transformation occurs in the substantial majority of adult cases.^2,3^ These differing characteristics also result in important differences in treatment philosophy for children compared to adults.^3^ For example, adjuvant chemoradiation has shown benefits in progression-free survival (PFS) and overall survival (OS) among LGG that occurs in patients >40 years and those <40 with subtotal resection (STR).^4^ By contrast, recent combined molecular and clinical analyses have identified pediatric LGG risk-stratified subgroups that differ in the potential benefit of adjuvant therapy.^5^ Furthermore, in pediatric LGG, radiation therapy has been shown to act as an independent adverse prognostic factor for OS.^6^ There is less observed heterogeneity in the clinical trajectory and treatment of high-grade glioma (HGG) between pediatric and adult populations.^7,8^

AYA, commonly defined as patients between 15 and 39 years of age, are a vulnerable subpopulation at the crossroads between pediatric and adult cohorts.^9,10^ National brain tumor registry data from the United States suggest that AYA glioma survival is more favorable than older adults (in whom HGG is more common), though survival rates are lower when compared to pediatric patients.^11^ However, AYA-specific prognostic and treatment data are rare due to overlapping inclusion in pediatric or adult cohorts combined with limited representation in clinical trials. Though it is now well accepted that glioma outcome varies by molecular alteration in both pediatric and adult cohorts, the molecular landscape of AYA glioma has not been well described, leading to a homogenous approach regardless of cancer genetics. This lack of AYA focus has consequences: mortality rates for AYA with CNS tumors have increased by 0.6% per year for males and 1% per year for females.^12^ Current literature is limited in defining the ideal treatment approach for this group. Thus, AYA patients treated in pediatric centers are most often treated according to pediatric guidelines, while those treated in adult centers are often treated with adult approaches.

Given the histological and molecular heterogeneity of glioma across the age spectrum, a rigorous evaluation of the available AYA glioma literature is required to inform patient counseling, therapeutic decisions, and future research priorities. Our objective was thus to review factors associated with survival outcomes in AYA glioma.

## Methods

Ethics approval was not required for this systematic review.

### Data Sources and Search Strategy

The review followed the Preferred Reporting Items for Systematic Reviews and Meta-Analyses (PRISMA) guidelines.^13^ Multiple databases including OVID MEDLINE, EMBASE and EBM Reviews-Cochrane library databases from inception to July 2020 were queried in collaboration with an academic librarian at the Hospital for Sick Children. A sample search strategy can be found in supplemental materials (Supplementary Table 1). Bibliographies of relevant reviews were further queried to ensure all relevant studies were captured.

#### Screening and search strategy

—Study inclusion criteria included: (1) original studies that reported predictors of cancer-related outcomes [eg, PFS, time to malignant progression (TtMP), OS]; (2) mean or median age at diagnosis within the AYA age range (15–39.9 years); (3) AYA patient sample size greater than 20; (4) diagnosis of glioma based on either WHO 2007 or WHO 2016 classification (Appendix 2); and (5) published in English between January 2010 and June 2020. Studies of pediatric and adult age groups were included if outcomes for AYA were reported separately, or if AYA patients represented more than 50% of the entire group. Exclusion criteria included low- and middle-income country studies (World Bank Definition), reviews, commentaries, editorials, conference abstracts, articles published before 2010, case series fewer than 20 patients, and studies using population-based mortality statistics.^14^

Abstracts were screened and assessed to identify pertinent studies (VZ). Full text review was conducted by two independent authors (VZ and AM). Discrepancies were reviewed by a third author when required (VK). The kappa coefficient was calculated to determine agreement between reviewers.

#### Data extraction and analysis

—The Checklist for critical Appraisal and data extraction for systematic Reviews of prediction Modeling Studies-Prognostic Factors (CHARMS-PF) was used to extract data from included texts.^15^ The following data were extracted from each study: study type, country of origin, sample size, mean/median age at diagnosis, length of follow-up, and all factors included in univariate or multivariable models of outcomes. Study quality was evaluated independently by two reviewers (AM and VK) utilizing the Quality In Prognosis Studies (QUIPS) tool to assess risk of bias.^15–18^ Six domains of possible bias were assessed through QUIPS: study participation, study attrition, prognostic factor measurement, outcome measurement, adjustment for other prognostic factors, and statistical analysis and reporting. Meta-analysis was not possible due to significant study heterogeneity. When comparing outcomes across studies, “event-free survival” was used to describe any outcome which incorporated disease progression, such as malignant progression-free survival (MPFS) or PFS. Studies’ definitions of malignant transformation and disease progression were heterogenous. A common definition for malignant transformation was pathological diagnosis of grade 3 or 4 glioma or imaging consistent with malignant transformation based on new or increased contrast enhancement and or the lesional growth pattern. Progression was commonly defined in studies by previously described response assessment frameworks such as Response Assessment in Neuro-Oncology (RANO).^19^ In instances where a p-value was reported without a hazard ratio or risk ratio, the primary source was examined, and the directionality of the effect was included in parentheses. Several figures were generated using the R Studio version 1.4.1717 and the *ggplot2* package.

## Results

The search strategy yielded 12 294 studies; removal of duplicates resulted in 10 336 unique studies. After abstract screening, 261 studies were identified as possibly meeting inclusion criteria and their full texts reviewed. Following full text review, 40 studies met inclusion criteria. Supplementary Figure 1 depicts the PRISMA workflow identifying included studies and reasons for exclusion. The kappa measure of agreement between reviewers for final study inclusion was 94.6% (95% CI 89.5–99.8%), or excellent.

### Study Characteristics

**Figure 1.**
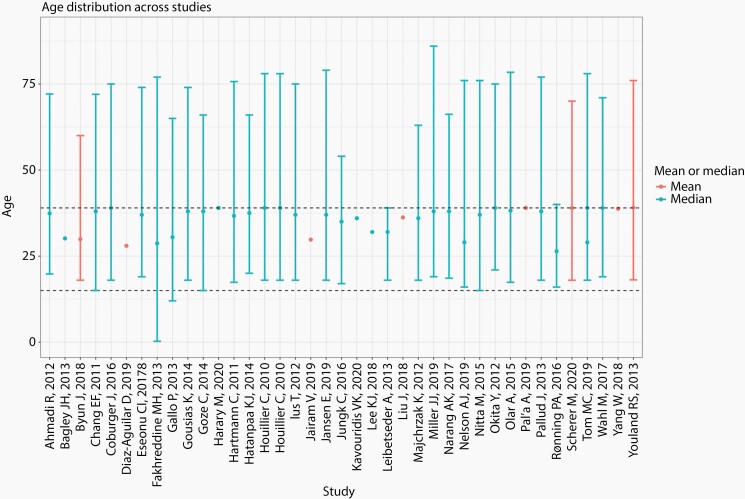
Mean and median age distributions of patients across studies. Dashed lines represent age range of adolescent and young adults (15–39). Vertical bars depict various study age ranges when available. .jpg file attached separately.

Forty studies met criteria for inclusion in the review: 39 studies were retrospective (single center, multi-center or national database studies) and 1 study was prospective. Countries of origin included: United States (*n* = 19), Germany (*n* = 8), France (*n* = 4), Italy (*n* = 2), Japan (*n* = 2), Poland (*n* = 1), Austria (*n* = 1), United Kingdom (*n* = 1), Norway (*n* = 1) and Korea (*n* = 1). There was substantial variability in sample size among studies, ranging from 25 to 3057 patients. Together, the studies represented 12 405 patients with an age range from 3 months to 86 years. Though greater than 50% of each study cohort was required to be AYA based on inclusion criteria, older adults and children were included in many studies as illustrated in Figure 1. There were three studies that specifically included spinal cord gliomas, 1 study that included both spinal cord and intracranial glioma and the remainder included intracranial glioma. Three studies did not provide OS for the overall cohort, while another 10 did not provide EFS. All studies included OS-based univariate or multivariable analyses.

### Overall Survival and Event-Free Survival

Glioma outcomes are summarized in Table 1. Two studies reported only on intracranial grade 1 glioma in which one showed an OS of 80% at 5 years and the other showed a reduced survival in the cohort undergoing external beam radiation therapy (EBRT) (< 60% 5 year OS) compared to those not undergoing adjuvant EBRT (> 75% 5 year OS).^20,21^ Two studies included combined cohorts of both grade 1 and 2 glioma in which OS ranged from 75.7 to 91.0% at 5 years.^22,23^ Twenty-six studies included grade 2 glioma only and reported 5-year OS ranging from 84 to 98%, with one study reporting 5-year OS of 69.2% in a subset of patients with radiographic velocity of diametric expansion over 8 mm/year.^24–49^ Among studies of grade 2 glioma, 5-year EFS ranged from 30 to 94%. Several studies included glioma subgroups across multiple pathological grades. 2 studies grouped grade 2 and 3 pleomorphic xantho-astrocytoma (PXA) with combined OS 76.3–89.5% at 5-years, 3 studies grouped grade 2 and 3 glioma together, 2 studies included grade 3 and 4 glioma, and 3 studies reported varying grades of spinal cord glioma, with 5-year OS ranging from 85.4% in grade 1 cases to 36.4% in grades 2, 3 and 4^50–59^ (Table 1).

**Table 1.** Summary table of overall survival and event-free survival data across included studies (*n* = 40)

Glioma type	First author, year of Publication	Country	Study design	Sample size	Glioma pathological subtype	Age at diagnosis (years)	Length of follow-up (months)	Overall survival	Event-free survival
Grade 1 glioma	Lee KJ 2018	US	Retrospective national cohort	3057	WHO Grade 1 astrocytoma (includes spinal cord)	Median 32	NOS	Patients undergoing EBRT 5 year < 60%, patients not undergoing EBRT5 year > 75%	NOS
	Nelson AJ 2019	UK	Retrospective single center	50	WHO Grade 1 glioma	Median 29 (16–76 range)	Median 3.5 years	5 year—80%	Median 7 years (95% CI 4.5–9.5)
Grade 1 and 2 glioma	Bagley JH 2013	US	Retrospective national cohort	166	Cerebellar WHO Grade 1 (*n* = 71) and Grade 2 (*n* = 95) astrocytoma	Median G1A 25.0 Median G2A 34.0	NOS	G1A: 5 year 91% 10 year 90% G2A: 5 year 69.5% 10 year 64%	NOS
	Rønning PA 2016	Norway	Retrospective national cohort	346	WHO Grade 1 and 2 glioma (female cohort) i) Pilocytic astrocytoma (*n* = 46) ii)Diffuse astrocytoma (*n* = 196) iii) Oligoastrocytoma (*n* = 26) iv) Oligodendroglioma (*n* = 78)	Median 26.4 (16–40 range)	Median 15.2 years	5 year—75.7% 10 year—54.8% G2 glioma cases only: Median 12.2 years (95% CI 10.7–17.5)	NOS
Grade 2 glioma	Ahmadi R 2012	Germany	Retrospective single center	100	Supratentorial WHO Grade 2 astrocytoma i) IDH1 mutant (*n* = 79) ii) IDH wt (*n* = 21)	Median 37.4 (19.8–72.1 range)	Median 81.1 (28–134.2 range)	Median 81.4 months (95% CI 5.5–274.8)	Median PFS 44.6 months (95% CI 1.0–267.0) Median TtMP 74.9 (95 % CI 1.6–236.2)
	Chang EF 2011	US	Retrospective single center	281	Infiltrative WHO Grade 2 gliomas i) Astrocytoma (*n* = 81) ii) Oligodendroglioma (*n* = 101) iii) Oligoastrocytoma (*n* = 99)	Median 38 (15–72 range)	Median 62.4 (3–152 range)	5 year 86%	5 year 62%
	Coburger J 2016	Germany	Retrospective multi-center	288	WHO Grade 2 gliomas i) Diffuse astrocytoma (*n* = 173) ii) Oligoastrocytoma (63) iii) Oligodendroglioma (*n* = 52)	Mean 39 (18–75 range)	Mean 52	Mean 21 months (95% CI 17–25)	Mean 68 months (95% CI 58–77) 5 year 94%
	Eseonu CI 2017	US	Retrospective single center	109	WHO Grade 2 gliomas i) Diffuse astrocytoma (*n* = 73) ii) Oligodendroglioma (*n* = 36)	Median 37 (19–74 range)	Median 62.4	5 year 84% 8 year 65%	5 year 70% 8 year 51%
	Gousias K 2014	Germany	Retrospective single center	148	WHO Grade 2 supratentorial glioma i) Diffuse astrocytoma (*n* = 76) ii) Oligoastrocytoma (*n* = 54) iii) Oligodendroglioma (*n* = 18)	Median 38 (18–74 range)	Median 59 (1–196 range)	5 year—86.1%*	Median PFS 70 months Median TtMP 98 months
	Goze C 2014	France	Retrospective multi-center	131	WHO Grade 2 supratentorial glioma i) Diffuse astrocytoma (*n* = 25) ii) Oligoastrocytoma (*n* = 71) iii) Oligodendroglioma (*n* = 35) a) 1p19q co-deleted (*n* = 38 out of 119 tested) b) P53 over-expression (*n* = 65 out of 125 tested) c) IDH1 mutant (*n* = 107 out of 131 tested)	Median 38 (15–66 range)	Median 55 (3.6–262 range)	82.4% survival at median observation period of 111 months	Median TtMP 51 months (42.7% of cohort in observed follow-up period)
	Harary M 2020	US	Retrospective national cohort	590	WHO Grade 2 oligodendroglioma (1p/19q-co-deleted)	Median 39 (29–52 IQR)	Median 41.5 (23.8–61.6 IQR)	Biopsy only: 5 year— 92.4% STR: 5 year—90.1% GTR: 5 year—96.5%	NOS
	Hartmann C 2011	Germany	Retrospective multi-center	89	WHO Grade 2 glioma i) Diffuse astrocytoma (*n* = 40) ii) Oligoastrocytoma (*n* = 23) iii) Oligodendroglioma (*n* = 26)	Median 36.7 (17.4–75.7 range)	Median 75.6	Median 15.5 years	Median 4.1 years (95% CI 3.1–5.1)
	Houillier C 2010	France	Retrospective multi-center	231	WHO Grade 2 glioma i) Diffuse astrocytoma (*n* = 43) ii) Oligoastrocytoma (*n* = 58) iii) Oligodendroglioma (*n* = 130)	Median 39 (18–78 range)	Median 95.1 (95% CI 82.5–107.3)	Median 175.8 months (95% CI 150.1–261)	Median 39.6 months (95% CI 35.8–44.5)
	Houillier C 2010	France	Retrospective single center	271	WHO Grade 2 glioma i) Diffuse astrocytoma (*n* = 47) ii) Oligoastrocytoma (*n* = 66) iii) Oligodendroglioma (*n* = 158)	Median 39 (18–78 range)	Median 69.2 (95% CI 60.3–78.7)	Median 133.3 months	Median 41.3 months
	Ius T 2012	Italy	Retrospective single center	190	WHO Grade 2 glioma supratentorial eloquent location i) Diffuse astrocytoma (*n* = 98) ii) Oligoastrocytoma (*n* = 34) iii) Oligodendroglioma (*n* = 58)	Median 37 (18–75 range)	Median 56.4 (4–155 range)	5 year—80% 8 year—66%	5 year—59% 8 year—35%
	Jairam V 2019	US	Retrospective national cohort	1032	WHO Grade 2 glioma i) Diffuse astrocytoma (*n* = 433) ii) Oligoastrocytoma (*n* = 256) iii) Oligodendroglioma (*n* = 343)	Mean 29.8 ± 6	Median 46.8	5 year—91.7%	NOS
	Jansen E 2019	Germany	Retrospective multi-center	110	WHO Grade 2 glioma i) Diffuse astrocytoma IDH mutant (*n* = 53) ii) Diffuse astrocytoma IDH wt (*n* = 18) iii) Oligodendroglioma (*n* = 39)	Median 37 (18–79 range)	Median 126 (95% CI 109–143)	5 year—88% 10 year—71% 15 year—57%	5 year—38% 10 year—18% 15 year—1%
	Jungk C 2016	Germany	Retrospective single center	46	WHO Grade 2 astrocytoma (*n* = 38 IDH1 mutated)	Median 35 (17–54 range)	Median 69 (17.5–164.6)	Median 119.8 months (17.5–164.6)	Median 45.1 (4.7–164.6)
	Kavouridis VK 2020	US	Retrospective single center	326	WHO Grade 2 glioma diffusely infiltrating i) Diffuse astrocytoma IDH mutant (*n* = 154) ii) Diffuse astrocytoma IDH wt (*n* = 32) iii) Oligodendroglioma (*n* = 140)	Median 36 (IQR 30–46)	Median 64.8 (31.2–114)	5 year—88.3% 10 year—70.1%	5 year—30.0% 10 year—12.7%
	Majchrzak K 2012	Poland	Prospective single center	68	WHO Grade 2 supratentorial glioma i) Diffuse astrocytoma (*n* = 46) ii) Oligoastrocytoma (*n* = 17) iii) Oligodendroglioma (*n* = 5)	Median 36 (18–63 range)	Median 34 (IQR 21–49)	5 year—91%	5 year—35%
	Narang AK 2017	US	Retrospective single center	108	WHO Grade 2 glioma with known radiologic progression (OS from date of progression)	Median 38 (18.6–66.2 range)	Median 131.1	Median 58.8 months 5 year—48%	Median 41.5 months
	Nitta M 2015	Japan	Retrospective single center	153	WHO Grade 2 supratentorial glioma i) Diffuse astrocytoma (*n* = 49) ii) Oligoastrocytoma (*n* = 45) iii) Oligodendroglioma (*n* = 59)	Median 37.0 (15–76 range)	NOS	5 year—95.1% 10 year—85.4%	Median 7.4 years
	Okita Y 2012	Japan	Retrospective single center	72	WHO Grade 2 glioma i) Diffuse astrocytoma (*n* = 49) ii) Oligoastrocytoma (*n* = 19) iii) Oligodendroglioma (*n* = 4)	Median 39.0 (21–75 range)	Median 6.4 years	Median 10.3 years	Median 5.8 years
	Pal’a A 2019	Germany	Retrospective multi-center	144	WHO Grade 2 diffuse glioma (IDH mutant only)	Mean 39 (± 11)	Median 6 years (4.8–6.3 95% CI)	5 year—97.6% Median 16.1 years	Median 3.9 years
	Pallud J 2013	France	Retrospective national cohort	407	WHO Grade 2 supratentorial glioma i) Velocity diametric expansion < 8 mm/ year (*n* = 335) ii) Velocity diametric expansion ≥ 8 mm/ year (*n* = 72)	Median 38.0 (18–77 range)	Median 73.0 (0–269)	Median 210 months (17–269) i) 5 year—92.8% ii) 5 year—69.2%a	Median TtMP 92 months (1–253) i) 5 year—73.4% ii) 5 year—27.7%
	Scherer M 2020	Germany	Retrospective multi-center	140	WHO Grade 2 glioma i) Diffuse astrocytoma (*n* = 92) ii) Oligodendroglioma (*n* = 48)	Mean 39.0 (18–70 range)	Median 62.0	Median 193 months (95% CI 141–245)	Median 43.0 months (95% CI 35–51)
	Tom MC 2019	US	Retrospective single center	486	WHO Grade 2 glioma i) IDH mut 1p19q co-deleted (*n* = 162) ii) IDH mut 1p19q intact (*n* = 125) iii) IDH wt (*n* = 185)	Median 39 (18–78 range)	Median 5.3 years (0.02–28.4)	5 year—82% i) IDH mut 1p19q co-deleted 5 year—94% ii) IDH mut 1p19q intact 5 year—89% iii) IDH wt 5 year—64%	5 year—86% malignant PFS
	Tom MC 2019	US	Retrospective single center	144	WHO Grade 2 glioma i) Diffuse astrocytoma (*n* =49) ii) Oligoastrocytoma (*n* = 36) iii) Oligodendroglioma (*n* = 59)	Median 29 (IQR 18–41)	Median 81 (IQR 36–132)	5 year—98% 10 year—90%	5 year—71% 10 year—53%
	Wahl M 2017	US	Prospective single center	120	WHO Grade 2 glioma i) Diffuse astrocytoma (*n* = 43) ii) Oligoastrocytoma (*n* = 20) iii) Oligodendroglioma (*n* = 57)	Median 39 (19–71 range)	Median 7.5 years	Median 9.7 years (95% CI 7.2–11.3)	Median 3.8 years (95% CI 3.0–5.0)
	Youland RS 2013	US	Retrospective single center	852	WHO Grade 2 glioma i) Diffuse astrocytoma (*n* = 293) ii) Oligoastrocytoma (*n* = 280) iii) Oligodendroglioma (*n* = 279)	Mean 39.1 (18.1–76.0)	Median 11.4 years (0.02–38.5)	Median 8.0 years	Median 4.4 years 10 year—22%
Grade 2 and 3 pleomorphic xantho- astrocytoma	Byun J 2018	Korea	Retrospective single center	25	WHO Grade 2 Pleomorphic xantho- astrocytoma (PXA) (*n* = 21) G3 PXA (*n* = 4)	Mean 29.9 (18–60 range)	Mean 51.4 (2–112 range)	G2 PXA: 5 year 89.5% 10 year 40.9% G3 PXA: 5 year 100% 10 year 0%	G2 PXA: 5 year 65.1% 7 year 52% G3 PXA: 5 year 0% 10 year 0%
	Gallo P 2013	Italy	Retrospective single center	40	WHO Grade 2 PXA (*n* = 32) G3 PXA (*n* = 8)	Median 30.5 (12–65 range)	Median 74	5 year—76.3% 10 year—68.2%	5 year—71.0% 10 year—58.0%
Grade 2 and 3 glioma	Hatanpaa KJ 2014	US	Retrospective single center	50	WHO Grade 2-III astrocytoma and oligoastrocytoma	Median 37.5 (20–66 range)	Median 51.6	NOS	NOS
	Miller JJ 2019	US	Retrospective single center	275	WHO Grade 2 (*n* = 134) and 3 glioma (*n* = 141) i) Oligodendroglioma (*n* = 95) ii) Astrocytoma (*n* = 180)	Median 38.0 (19–86 range)	Median 6.4 years	Median 18.7 years (95% CI 12.2-not reached)	Median 5.7 years (95% CI 4.7–6.4)
	Olar A 2015	US	Retrospective multi-center	558	WHO Grade 2 and 3 diffuse glioma i) Grade 2 (*n* = 262) ii) Grade 3 (*n* = 296)	Median 38.2 (17.4–78.4 range)	Median 7.4 years	G2 glioma: median 12.41 years G3 glioma: Median 13.35 years	NOS
High-grade glioma (Grade 3 and 4)	Yang W 2018	US	Retrospective national cohort	353	Peri-ventricular or subventricular zone Grade 3 and Grade 4 glioma i) Glioblastoma (*n* = 172) ii) Anaplastic ependymoma (*n* = 70) iii) Anaplastic astrocytoma (*n* = 65) iv) Other (*n* = 46)	Mean 38.77 (± 24.95)	NOS	Median 12 months (95% CI 10–15)	NOS
	Leibetseder A 2013	Austria	Retrospective multi-center	47	WHO Grade 4 astrocytoma	Median 32 (18–39 range)	NOS	Median 28 months (95% CI 24–31.6)	Median 12 months (95% CI 9.5–14)
Spinal cord glioma	Diaz-Aguilar D 2019	US	Retrospective national cohort	561	WHO Grade 1 and 2 gliomas spinal cord i) Pilocytic astrocytoma (*n* = 247) ii) Diffuse astrocytoma (*n* = 64) iii) Astrocytoma NOS (*n* = 222) iv) Glioma NOS (*n* = 28)	Mean 28 (± 22)	NOS	NOS	NOS
	Fakhreddine MH 2013	US	Retrospective single center	83	Spinal cord astrocytoma i) WHO Grade 1 (*n* = 31) ii) WHO Grade 2 (*n* = 14) iii) WHO Grade 3 (*n* = 18) iv) WHO Grade 4 (*n* = 18) v) Indeterminate either Grade 3 or IV (*n* = 2)	Median 28.7 (0.25–77 range)	Median 49.2	G1A: 5 year 85.4% Infiltrative astrocytoma (G2A, G3A and G4A): 5 year 36.4%	G1A: Median 3.33 years Infiltrative astrocytoma (G2A, G3A and G4A): Pooled median 0.89 years
	Liu J 2018	US	Retrospective national cohort	158	WHO Grade 3 and IV spinal cord glioma i) Anaplastic astrocytoma (*n* = 14) ii) Anaplastic ependymoma (*n* = 14) iii) Glioblastoma (*n* = 111)	Mean 36.23 (± 21.0)	NOS	Median 20 months (9–42.75)	NOS

Pooled follow-up, median/mean age, OS and PFS when available unless reported separately in original article.

NOS, not otherwise specified; G1A, Grade 1 astrocytoma; G2A, Grade 2 astrocytoma; G3A, Grade 3 astrocytoma; EBRT, external beam radiation therapy.

*Limited number of patients died during follow-up therefore robust multivariate OS modeling was not possible.

### Patient Factors

Several patient factors were associated with superior OS and EFS across glioma grade following adjusted multivariable analysis (Tables 2–4). Increased age was often associated with worse OS when age was evaluated as a continuous variable,^20,23,34,38,58,59^ including cohorts of pilocytic astrocytoma alone, combined grade 1 and 2 gliomas, combined grade 2 and 3 gliomas, and of peri-ventricular HGG. Within the AYA group, the following younger age clusters were associated with improved OS: age <18 years,^51^ age <30 years,^53^ and age <40 years.^22,42^ Only one study showed a negative impact of age younger than 40 on OS.^46^ Several studies in contrast did not find a significant association between age and OS in multivariable analysis.^30–33,36,43,46,52,54^ Three studies demonstrated that younger age was associated with improved EFS.^45,47,53^

**Table 2.** Demographic, radiographic, tumor and treatment influences on AYA WHO Grade 1 glioma event-free survival (EFS) and overall survival (OS)

Imaging, treatment and tumor factors		Study	Overall survival		Study	Event-free survival	
			Univariate	Multivariate		Univariate	Multivariate
Demographic factors	Age (continuous)	Rønning PA, 2016	**HR = 1.067, *P* < .001**	**HR = 1.049, *P* < .001**			
		Lee KJ, 2018	***P* < .001**	**HR = 1.050, *P* < .001**			
	Age ≥ 40	Bagley JH, 2013		**HR = 7.30, *P* < .0001**			
	Age 0–18 (ref.) vs. i) 18–65 ii) > 65	Diaz-Aguilar D, 2019	***P* < .001**	i) **HR = 3.05, *P* = .024** ii) **HR = 5.26, *P* < .001**			
	Female sex	Bagley JH, 2013		**HR = 0.28, *P* < .001**			
	Median annual income < $38 000 (ref.) vs. i) $38 000–$47 999 ii) $48 000–$62 999 iii) > $63 000	Lee KJ, 2018	***P* = .01**	i) **HR = 0.621, *P* = .001** ii) **HR = 0.543, *P* < .001** iii) **HR = 0.600, *P* < .001**			
	Charlson-Deyo Comorbidity index = 0 (ref.) vs. i) 1 ii) 2	Lee KJ, 2018	***P* < .001**	i) NS ii) **HR = 1.647, *P* = .009**			
Radiographic characteristics	Tumor size 1–19 mm (ref.) vs. i) 20–39 mm ii) 40–59 mm iii) 60–79 mm iv) 80–99 mm v) 100+ mm	Lee KJ, 2018	***P* < .001**	i) **HR = 1.661, *P* = .010** ii) **HR = 1.803, *P* = .006** iii) **HR = 3.029, *P* < .001** iv) NS v) NS			
	Location of tumor supratentorial (ref.) vs. infratentorial and spinal cord	Lee KJ, 2018	Supratentorial superior ***P* = .01**	NS			
Tumor presentation	Spinal astrocytoma motor deficit i) G1A cohort ii) Infiltrative cohort (G2A, G3A, G4A)				Fakhreddine MH, 2013	i) Motor deficit superior ***P* = .040** ii) NS	
	Spinal astrocytoma symptoms ≥ 4.6 months i) G1A cohort ii) Infiltrative cohort (G2A, G3A, G4A)	Fakhreddine MH, 2013	i) NS ii) Symptoms ≥ 4.6 months superior ***P* = .027**	NS			
	Spinal astrocytoma motor deficit i) G1A cohort ii) Infiltrative cohort (G2A, G3A, G4A)				Fakhreddine MH, 2013	i) Motor deficit superior ***P* = .040** ii) NS	
Histolological factors	G1 (ref.) vs G2 astrocytoma	Bagley JH, 2013		**HR = 2.76, *P* = .028**			
	Spinal cord G1 (ref.) vs. G2 astrocytoma	Diaz-Aguilar D, 2019	**HR = 2.34, *P* < .001**	NS			
	Diffuse astrocytoma (ref.) vs. i) Oligoastrocytoma ii) Oligodendroglioma iii) Pilocytic astrocytoma	Rønning PA, 2016	i) NS ii) NS iii) **0.251, *P* < .001**	i) NS ii) NS iii) **0.380, *P* < .05**			
Chemotherapy	Spinal astrocytoma adjuvant chemotherapy i) G1A cohort ii) Infiltrative cohort (G2A, G3A, G4A)	Fakhreddine MH, 2013	i) Adjuvant chemotherapy superior ***P* = .032** ii) NS	ii) NS	Fakhreddine MH, 2013	i) ***P* = .023** ii) NS	ii) **HR = 0.22, *P* = .0075**
Radiation therapy	G1 and G2 glioma post-operative radiotherapy	Rønning PA, 2016	**HR = 2.013, *P* < .001**	**HR = 1.808, *P* < .01**			
	Radiation technique no radiation (ref.) vs. i) EBRT ii) Stereotactic radiosurgery iii) Radiation NOS	Lee KJ, 2018	***P* < .001**	i) **HR = 3.370, *P* < .001** ii) NS iii) NS			
	Spinal cord G1 and G2 glioma post-operative radiotherapy	Diaz-Aguilar D, 2019	***P* < .001**	**HR = 2.78, *P* < .001**			
	Spinal cord astrocytoma post-operative radiotherapy i) G1A cohort ii) Infiltrative cohort (G2A, G3A, G4A)				Fakhreddine MH, 2013	i) ***P* = .047** (worsened EFS in radiated group) ii) NS	NS (all astrocytoma grades pooled in multivariate analysis)
Surgical factors	G1 and G2 spinal cord glioma no surgery (ref.) vs. i) STR ii) GTR	Diaz-Aguilar D, 2019	***P* < .001**	i) NS ii) **HR = 0.38, *P* = .027**			
	G1 and G2 glioma biopsy (ref.) vs. resection	Rønning PA, 2016	**HR = 0.544, *P* < .01**	NS			
	Biopsy alone (ref.) vs. i) < 25% residual following STR ii) > 25% residual following STR				Nelson AJ, 2019	i) Biopsy inferior ***P* = .022** ii) Biopsy inferior ***P* = .005**	

Cases with pooled WHO Grade 2 gliomas were included if they included WHO Grade 1 lesions.

NS, not significant; KPS, Karnofsky Performance Status; HR, Hazard ratio.

Significant *P*-values without indication of effect directionality (absence of reported hazard ratio) contain a note about superior or inferior effect on OS or EFS.

Bolded fields indicate statistical significance of the variable in cited study at an alpha of 0.05.

**Table 4.** Demographic, radiographic, tumor and treatment influences on AYA WHO Grade 3 and 4 glioma event-free survival (EFS) and overall survival (OS)

Imaging, treatment and tumor factors		Study	Overall survival		Study	Event-free survival	
			Univariate	Multivariate		Univariate	Multivariate
Demographic factors	Age (continuous)	Yang W, 2018	***P* < .001**	**HR = 1.19, *P* < .001**			
		Olar A, 2015		**HR = 1.03, *P* < .0001**			
	Age ≤ 30	Gallo P, 2013	**HR = 0.81, *P* = .024**	**HR = 0.05, *P* = .01**	Gallo P, 2013	NS	**HR = 0.15, *P* = .01**
		Leibetseder A, 2013	Age ≤ 30 superior ***P* < .05**				
	Female sex	Hatanpaa KJ, 2014		**RR = 5.02, *P* = .022**			
Radiographic characteristics	G3 and G4 spinal cord glioma tumor extension (ref. localized) i) Regional extension ii) Invasive/distal extension iii) Unknown	Liu J, 2018	i) NS ii) NS iii) **HR = 1.68, *P* = .045**				
Histological factors	G2 (ref.) vs G3 PXA	Gallo P, 2013		**HR = 12.58, *P* = .003**			
	G2 and G3 glioma oligodendroglioma (ref.) vs. astrocytoma	Miller JJ, 2019	Oligodendroglioma superior ***P* = .025**				
	Spinal astrocytoma G2A (ref.) vs. G3A vs. G4A	Fakhreddine MH, 2013	***P* = .0004**	**HR = 6.56** (G3A) and **HR = 14.7** (G4A), ***P* = .014**			
	Recurrent G2 glioma new Histological grade unchanged vs. malignant degeneration (G3 or G4 glioma)	Narang AK, 2017	***P* < .001**	**HR = 4.24, *P* = .001**			
Molecular factors	G2 and G3 glioma IDH mutant 1p19q co-deletion (ref.) vs. other	Olar A, 2015	1p19q co-deletion superior ***P* < .0001**				
	G2 and G3 glioma 1p19q status non co-deleted (ref.) vs. co-deleted	Olar A, 2015		**HR = 0.53, *P* = .0265**			
	G2 and G3 glioma IDH mutant (ref.) vs. wt	Hatanpaa KJ, 2014	***P* = .0006**	**RR = 6.99, *P* = .0035**			
		Miller JJ, 2019	IDH mutant superior ***P* = .015**				
	G2 and G3 glioma IDH wt (ref.) vs. mutant	Olar A, 2015	NS	**HR = 0.38, *P* < .0001**			
	G2 and G3 glioma nestin level (continuous)	Hatanpaa KJ, 2014	***P* = .0022**	**RR=13.42, *P* = .0004**			
	G2 and G3 glioma mitotic index >4% i) IDH mutant ii) IDH wt	Olar A, 2015		**HR = 1.70, *P* < .0001** i) NS ii) **HR = 2.73, *P* = .0010**			
Chemotherapy	G2 and G3 glioma adjuvant chemotherapy only				Miller JJ, 2019	**HR = 1.6, *P* = .047**	NS
	G2 and G3 glioma combined adjuvant chemoradiation				Miller JJ, 2019	**HR = 0.57, *P* = .0026**	**HR = 0.38, *P* = .0002**
Radiation therapy	G2 and G3 glioma adjuvant radiotherapy i) IDH mutant ii) IDH wt	Olar A, 2015	–	**HR = 0.58, *P* = .0020** i) **HR = 0.55, *P* = .0028** ii) NS	Miller JJ, 2019	**HR = 0.54, *P* = .013**	**HR = 0.35, *P* = .000147** (no mutational status in analysis)
	G3 and G4 spinal cord glioma post- operative radiotherapy	Liu J, 2018	NS	**HR = 0.54, *P* = .031**			
	G3 and G4 peri-ventricular glioma adjuvant radiotherapy	Yang W, 2018	**HR = 0.55, *P* < .001**	**HR = 0.50, *P* < .001**			
Surgical factors	G2 and G3 glioma GTR (ref.) vs. STR	Hatanpaa KJ, 2014		**RR = 3.97, *P* = .037**			
	Recurrent G2 glioma with transformation to G3 or G4 histology: STR or biopsy (ref.) vs. GTR or NTR	Narang AK, 2017	***P* = .02**	**HR = 0.36, *P* = .001**			
	G3 and G4 peri-ventricular glioma no resection (ref.) vs. i) Biopsy ii) STR iii) GTR	Yang W, 2018	i) NS ii) **HR = 0.62, *P* = .007** iii) **HR = 0.45, *P* < .001**	i) NS ii) NSi iii) NS			

Cases with pooled WHO Grade 2 gliomas were included if they included WHO Grade 3 lesions.

Bolded fields indicate statistical significance of the variable in cited study at an alpha of 0.05.

The relationship between sex and OS and EFS was conflicting with no clear prognostic effect.^22,29,32,38,46,54^ Three studies showed no effect of patient sex on OS.^30,33,39,53^ Other patient-related factors associated with favorable OS included private health insurance in a United States cohort,^30^ median annual income greater than $38 000,^20^ Charles-Deyo Comorbidity Index score of 0 vs. 2,^20^ and Karnofsky Performance Score (KPS) greater than 80.^32,33,42^ KPS over 80 was associated with favorable EFS in 1 study following multivariable analysis,^42^ and though KPS was significantly associated with EFS in univariate analysis in three additional studies, it lost significance when adjusted for other factors.^32,33,47^

### Disease and Treatment-Related Factors

#### Grade 1 glioma

—Several disease and treatment-related factors were significantly associated with OS and EFS among patients with grade 1 glioma or studies combining grade 1 and 2 gliomas (Table 2). Pre-operative lesion size over 19 mm^20^ and grade 2 compared to grade 1 histology^22,23^ were associated with inferior OS, while location of tumor in the supratentorial compartment was favorable compared to spinal cord or infratentorial locations following univariate analysis, though non-significant after multivariable analysis (though brainstem lesion inclusion in the infratentorial category may have biased this finding).^20^ Symptom duration in spinal cord glioma was not significantly associated with OS after multivariable analysis.^52^ Treatment-related factors positively influencing OS included gross-total resection (GTR) in spinal cord glioma cases.^51^

Three studies found adjuvant radiation to be associated with inferior OS even after adjustment for other factors.^20,23^ The first study by Lee et al. examined a national cohort of patients with pilocytic astrocytoma and adjusted for age, median income, tumor volume and comorbidity scores. They found adjuvant external beam radiotherapy (EBRT) was associated with a significantly worsened OS compared to no radiotherapy (patients undergoing EBRT 5-year OS < 60% compared to ≥ 75% 5-year OS in patients receiving other therapies).^20^ The same study showed a trend towards inferior OS, though non-significant, when stereotactic radiotherapy was compared to no radiotherapy.^20^ The authors nonetheless attributed their finding to confounding by other important factors including eloquent location and tumor resectability. The second study examined the effect of pregnancy on LGG survival.^23^ They showed that post-operative radiation therapy was associated with significantly inferior OS in combined grade 1 and 2 gliomas as well as grade 2 gliomas alone following multivariable adjustment, though the authors did not provide a list of what variables were adjusted for. The third study, examining low-grade spinal cord glioma, demonstrated a negative association between adjuvant radiotherapy and OS following adjustment for grade, age and surgical history.^51^

#### Grade 2 glioma

—Radiographic factors associated with OS and EFS among patients with grade 2 gliomas are summarized in Table 3. Imaging-related factors negatively associated with OS following multivariable analysis included: eloquent location,^25^ tumor volume over 100 cm^3,29,44^ larger tumor size as a continuous variable,^38^ velocity of diametric expansion over 8 mm/year,^29,44^ size greater than 5 cm^35,49^ and size greater than 6 cm.^30^ Factors initially significantly associated with OS in univariate analyses but which lost association in multivariable analyses included contrast enhancement on MRI^29,40^ and corpus callosum involvement.^29^ There was significant negative influence of eloquent location,^25^ MRI contrast enhancement,^28,44^ tumor volume greater than 100 cm^3,44^ tumor size as a continuous variable,^38,45,47^ diametric annual expansion greater than 8 mm,^29,44^ size greater than 5 cm^41,46,49^ and parietal compared to frontal location^29^ on grade 2 glioma EFS following adjusted multivariable analysis.

**Table 3.** Demographic and radiographic factors associated with AYA WHO Grade 2 glioma event-free survival (EFS) and overall survival (OS)

Demographic and radiographic factors		Study	Overall survival		Study	Event-free survival	
			Univariate	Multivariate		Univariate	Multivariate
Age	Age (continuous)	Eseonu CI, 2017	**HR = 1.098, *P* = .03**		Tom MC,2019	***P* = .005**	**HR = 1.05, *P* = .03**
		Ius T, 2012	**HR = 1.030, *P* = .011**	**HR = 1.035, *P* = .003**			
		Kavouridis VK, 2020		**HR = 1.06, *P* < .001**			
		Majchrzak K, 2012	**HR = 1.12, *P* = .032**				
	Age ≤ 40	Okita Y, 2012	***P* = .04**	**HR = 0.400, *P* = .02**	Scherer M, 2020		**HR = 0.60, *P* = .03**
	Age ≥ 40	Jansen E, 2019	Age ≥ 40 inferior ***P* = .048**				
		Youland RS, 2013		**HR = 1.36, *P* = .001**			
		Tom MC, 2019	**-*P* < .001**	**HR = 2.2, *P* < .001**			
	Age ≥ 50	Nitta M, 2015	**HR = 5.43, *P* = .0089**	**HR = 5.38, *P* = .0121**			
	Age > 55	Houillier C 2010	Age > 55 inferior ***P* = 0.001**	NS			
Sex	Male sex	Goze C, 2014	**HR = 5.06, *P* = .002**	**HR = 10.22, *P* = .001**	Tom MC,2019	***P* = .009**	**HR = 2.1, *P* = .009**
		Kavouridis VK, 2020		**HR = 2.02, *P* = .042**			
		Tom MC, 2019	***P* = .003**	**HR = 1.7, *P* = .002**			
	Female sex	Houillier C, 2010	***P* = .04**	**HR = 0.45, *P* = .01**			
		Houillier C 2010	Female sex superior ***P* = .01**	NS			
Financial status	Non-insured (ref.) vs. i) Private insurance ii) Medicare	Harary M, 2020	–	i) **HR = 0.24, *P* = .04** ii) NS			
	Median annual income < $38 000	Jairam V, 2019	**HR = 1.88, *P* = .043**				
Functional status	KPS (continuous)	Ahmadi R, 2012	Higher KPS superior ***P* = .0004**		Ahmadi R, 2012	Higher KPS superior ***P* = .0009**	
		Gousias K, 2014	**HR = 0.136, *P* < .001**				
					Tom MC,2019	**HR = 0.97, *P* = .045**	NS
	KPS ≥ 90	Gousias K, 2014	**HR = 0.136, *P* < .001**		Gousias K, 2014	**HR = 0.441, *P* = .001**	
	KPS > 80	Houillier C, 2010	***P* = .001**	**HR = 0.40, *P* = .009**	Houillier C, 2010	KPS ≥ 90 superior ***P* = .03**	NS
		Houillier C 2010	***P* < .0001**	**HR = 0.21, *P* = .0003**	Houillier C 2010	KPS > 80 superior ***P* = .01**	NS
		Okita Y, 2012	***P* = .0006**	**HR = 0.045, *P* = .0002**	Okita Y, 2012	***P* = .01**	**HR = 0.179, *P* = .01**
Radiographic factors	G2 glioma eloquent location	Chang EF, 2011	***P* < .0001**	**HR = 6.1, *P* < .001**	Chang EF, 2011	***P* < .0001**	**HR = 1.9, *P* = .003**
		Gousias K, 2014	**HR = 3.498, *P* = .008**		Gousias K, 2014	Eloquent location inferior ***P* < .001**	
	False eloquent group (ref.) vs. true eloquent group by intra-operative mapping*	Chang EF, 2011	False eloquent group superior ***P* < .001**				
	G2 glioma MRI contrast enhancement	Goze C, 2014	**HR = 1.79, *P* = .001**	NS	Gousias K, 2014	**HR = 2.335, *P* = 0.013**	**HR = 2.441, *P* = .012**
		Narang AK, 2017^2^	Contrast enhancement inferior ***P* = .03** (recurrent cases)	NS	Pallud J, 2013	***P* = .014**	**HR = 1.44, *P* < .011**
	G2 glioma corpus callosum involvement	Goze C, 2014	**HR = 4.69, *P* = .042**	NS	Pallud J, 2013	**HR = 1.73, *P* = .003**	
	G2 glioma tumor volume ≥ 100 cm^3^	Goze C, 2014	**HR = 2.44, *P* = .0022**	**HR = 9.69, *P* = .017**	Goze C, 2014	**HR = 2.44, *P* = .022**	NS
		Pallud J, 2013	**HR = 2.31, *P* = .002**	**HR = 2.92, *P* = .001**	Pallud J, 2013	***P* = .001**	**HR = 1.76, *P* = .008**
	G2 glioma tumor size/ volume (continuous)	Ius T, 2012	**HR = 8.20, *P* < .0001**		Ius T, 2012	**HR = 3.256, *P* = .001**	
		Kavouridis VK, 2020		**HR = 1.01, *P* = .016**	Tom MC,2019	**HR = 1.06, *P* < .0001**	**HR = 1.07, *P* < .0001**
					Kavouridis VK, 2020		**HR = 1.00, *P* = .009**
					Majchrzak K, 2012	**HR = 1.01, *P* = .005**	
					Scherer M, 2020		**HR = 1.007, *P* = .02**
	G2 glioma velocity of diametric expansion ≥ 8 mm/year	Goze C, 2014	**HR = 6.61, *P* < .0001**	**HR = 26.3, *P* < .0001**	Goze C, 2014	**HR = 4.18, *P* < .0001**	**HR = 4.23, *P* = .001**
		Pallud J, 2013	**HR = 3.96, *P* < .001**	**HR = 4.62, *P* < .001**	Pallud J, 2013	**HR = 3.50, *P* < .001**	**HR = 3.87, *P* < .001**
	G2 glioma > 5 cm	Jairam V, 2019	**HR = 2.27, *P* = .010**	**HR = 1.95, *P* = .03**	Nitta M, 2015	NS	**HR = 1.89, *P* = .0428**
		Tom MC, 2019	Glioma > 5 cm inferior ***P* = .05**		Tom MC, 2019	***P* < .001**	**HR = 3.5, *P* < .001**
		Youland RS, 2013		**HR = 1.70, *P* < .0001**	Youland RS, 2013		**HR = 1.85, *P* < .0001**
	G2 glioma > 3 cm				Gousias K, 2014	Size > 3 cm inferior ***P* = .006**	
	G2 oligodendroglioma tumor size (ref. 2.1–4 cm) i) ≤ 2 cm ii) 4.1–6 cm iii) > 6 cm	Harary M, 2020		i) NS ii) NS iii) **HR = 4.56, *P* = .02**			
	G2 glioma relative cerebral blood volume measurements	Majchrzak K, 2012	**HR = 7.39, *P* = .002**		Majchrzak K, 2012	**HR = 1.70, *P* = .033**	
	G2 glioma anatomic location (frontal lobe ref.) i) Temporal ii) Parietal iii) Insular				Goze C, 2014	i) NS ii) NS iii) NS	i) NS ii) **HR = 4.20, *P* = .019** iii) NS
	G2 astrocytoma volumetric difference between T2 FLAIR signal and T1W signal on pre-operative Imaging (continuous)				Jungk C, 2016	**HR 1.03, *P* = .028**	

*Patients within the group of presumed eloquent low-grade gliomas underwent intra-operative mapping. Positive intra-operative mapping cases were deemed *true eloquent* and those with negative intra-operative mapping were deemed *false eloquent.*

Bolded fields indicate statistical significance of the variable in cited study at an alpha of 0.05

Histological and molecular factors are shown in Table 5. Among patients with astrocytomas, grade 2 histology conferred significantly worse OS than grade 1 histology.^22^ Diffuse astrocytoma histology was associated with inferior OS compared to oligoastrocytoma or oligodendroglioma histology following multivariable analysis.^34–36,41,42,49^ Oligodendroglioma was variably defined either histologically or molecularly across articles. Oligodendroglioma showed significantly favorable OS compared to IDH mutant and IDH wildtype astrocytoma.^38,46^ IDH mutant status^29,33,37,42^ and 1p19q co-deletion^32,33^ were positively associated with longer EFS. In one cohort of diffuse supratentorial low-grade gliomas, 1p19q co-deletion status was non-significant after adjusted multivariable analysis.^29^ In multivariable analysis, EFS was significantly inferior among those with diffuse astrocytoma histology,^34,49^ adjusted for IDH mutational status.^46^ IDH mutant status,^29^ 1p19q co-deletion^32,33^ and O6-methylguanine-DNA methyl-transferase (MGMT) methylation^33^ were favorably associated with prolonged EFS when compared to IDH wild type gliomas. Diffuse astrocytic histology^43,47^ and p53 over-expression^47^ were significantly negatively associated with EFS in univariate analysis but after adjustment in multivariable analysis were no longer significant. Notably, the studies that described IDH mutational status and influence on prognosis all comprised of cohorts that despite meeting our inclusion criteria, included substantial numbers of older adults (Figure 1). For example, of the 26 studies that included AYA patients with grade 2 glioma, 24 had a mean or median age above 30.

**Table 5.** Molecular and Histological influences on AYA WHO Grade 2 glioma event-free survival (EFS) and overall survival (OS)

Histological and molecular factors	Study	Overall survival		Study	Event-free survival	
		Univariate	Multivariate		Univariate	Multivariate
G1 (ref.) vs G2 astrocytoma	Bagley JH, 2013		**HR = 2.76, *P* = .028**			
Non-oligodendroglioma histology and tumor size > 5 cm after surgery (ref.) vs. all other groups	Jairam V, 2019	**HR = 3.04, *P* < .001**				
G2 glioma oligodendroglioma or oligoastrocytoma (ref.) vs. diffuse astrocytoma	Wahl M, 2017	Oligodendroglioma superior ***P* = .007**		Ius T, 2012	**HR = 2.273, *P* = .003**	
	Ius T, 2012		**HR = 2.974, *P* = .005**	Nitta M, 2015	**HR = 2.08, *P* = .0140**	**HR = 1.86, *P* = .0485**
	Jairam V, 2019	**HR = 2.69, *P* = .002**	**HR = 2.50, *P* = .02**	Tom MC,2019	**HR = 2.21, *P* = .02**	NS
	Youland RS, 2013		**HR = 1.60, *P* < .0001**	Youland RS, 2013		**HR = 1.29, *P* = .007**
	Ius T, 2012	**HR = 4.262, *P* = .001**				
	Nitta M, 2015	**HR = 4.98, *P* = .0143**	**HR = 5.23, *P* = .0172**			
G2 glioma diffuse astrocytoma (ref.) vs. oligodendroglioma	Okita Y, 2012	***P* = .04**	**HR = 0.290, *P* = .02**	Houillier C, 2010	Oligodendroglioma superior ***P* = .03**	
	Jansen E, 2019	***P* = .002**	HR = 0.286*, *P* = .001	Pal’a A, 2019	Oligodendroglioma superior ***P* = .026**	NS
G2 glioma oligodendroglioma (ref.) vs. oligoastrocytoma				Tom MC,2019	**HR = 2.28, *P* = .03**	**HR = 3.13, *P* = .05**
G2 glioma oligodendroglioma (ref.) vs. i) IDH mutant astrocytoma ii) IDH wt astrocytoma	Kavouridis VK, 2020	–	i) **HR = 7.76, *P* < .001** ii) **HR = 20.6, *P* < .001**	Kavouridis VK, 2020	–	i) **HR = 1.98, *P* < .001** ii) NS
	Wahl M, 2017	Oligodendroglioma superior ***P* = .01**		Wahl M, 2017	Oligodendroglioma superior ***P* < .001**	
	Tom MC, 2019	i) NS ii) ***P* = .001**	i) **HR = 2.3, *P* = .001** ii) **HR = 2.9, *P* < .001**	Tom MC,2019	i) NS ii) ***P* = .05**	i) **HR = 2.7, *P* = .009** ii) **HR = 5.5, *P* < .001**
G2 glioma IDH wt (ref.) vs. IDH1/2 mutant	Jungk C, 2016	**HR = 0.11, *P* = .0003**	**HR = 0.091, *P* = .002**			
	Houillier C, 2010	***P* = .002**	**HR = 0.32, *P* = .003**			
	Okita Y, 2012	***P* = .004**	**HR = 0.365, *P* = .01**			
	Goze C, 2014	HR = 0.306*, *P* = .044	HR = 0.056*, *P* = .007	Tom MC,2019	HR = 0.199*, *P* < .0001	**HR = 0.314, *P* = .025**
G2 glioma 1p19q co-deletion (ref. non co-deleted)	Houillier C, 2010	***P* < .0001**	**HR = 0.16, *P* = .0001**	Houillier C, 2010	***P* = .002**	**HR = 0.50, *P* = .0006**
	Houillier C, 2010	***P* = .0001**	**HR = 0.3, *P* = .004**	Houillier C 2010	***P* = .002**	**HR = 0.6, *P* = .04**
	Eseonu CI, 2017	**HR = 0.291, *P* = .05**		Youland RS, 2013	1p19q co-deletion superior ***P* < .0001**	
	Pallud J, 2013	**HR = 0.45, *P* = .040**				
	Youland RS, 2013	1p19q co-deletion superior ***P* = .0001**				
	Goze C, 2014	HR = 0.256*, *P* = .031	NS			
G2 glioma p53 over-expression (>10%)				Houillier C 2010	P53 over-expression inferior ***P* = .02**	
				Tom MC,2019	**HR = 2.43, *P* = .01**	NS
MGMT promoter non- methylation				Houillier C 2010	***P* = .001**	**HR = 2.3, *P* = .02**

*Inverse hazard ratios were reported to compile into common categories.

Bolded fields indicate statistical significance of the variable in cited study at an alpha of 0.05.

Treatment-related variables are summarized in Table 6. The impact of adjuvant chemoradiotherapy on OS and EFS was mixed. Combined adjuvant chemotherapy and radiotherapy positively impacted OS and EFS among grade 2 glioma patients in one study compared to adjuvant radiotherapy alone following multivariable analysis.^42^ Within this study the effect of adjuvant chemoradiotherapy was most pronounced in cases of IDH 1/2 mutant cases. By contrast Pal’a et al^43^ examined only IDH mutant grade 2 glioma patients and found a negative impact of adjuvant chemoradiotherapy on EFS and OS after adjusting for age over 40 years, extent of resection, recurrent surgery and histology. Coburger et al^26^ also showed a negative impact of adjuvant chemoradiotherapy compared to no adjuvant therapy on EFS in a cohort of grade 2 glioma after adjusting for age, recurrent surgery, histology and residual tumor in their multivariable model. One group showed in LGG that combined chemoradiotherapy (temozolomide) was superior in EFS compared to chemotherapy alone in a multivariable model with covariates gender, tumor size, molecular characteristics and adjuvant therapy regimen.^46^

**Table 6.** Treatment-related influences on AYA WHO Grade 2 glioma event-free survival (EFS) and overall survival (OS)

Treatment factors		Study	Overall survival		Study	Event-free survival	
			Univariate	Multivariate		Univariate	Multivariate
Combined adjuvant therapy	G2 glioma post-operative radiotherapy alone (ref.) vs. chemoradiotherapy	Okita Y, 2012	***P* = .0002**	**HR = 0.198, *P* = .002**	Okita Y, 2012	***P* = .01**	**HR = 0.408, *P* = .04**
	G2 glioma IDH mutant adjuvant therapy (yes ref. vs. no) i) No therapy vs. chemotherapy ii) No therapy vs. radiotherapy iii) No therapy vs. chemoradiotherapy	Pal’a A, 2019	No adjuvant therapy superior ***P* = .003**	No adjuvant therapy superior ***P* = .009** i) NS ii) NS iii) **HR = 20.175, *P* = .001**	Pal’a A, 2019	No adjuvant therapy superior ***P* = .003**	**HR not stated** ***P* = .030** i) NS ii) NS iii) **HR = 2.745, *P* = .004**
	G2 glioma temozolomide and radiotherapy (ref.) vs. i) Observation ii) Radiation alone iii) Temozolomide alone	Tom MC, 2019	i) **HR = 0.3, *P* < .001** ii) NS iii) **HR = 0.4, *P* = .004**		Tom MC, 2019	i) NS ii) NS iii) NS	i) NS ii) NS iii) **HR = 3.8, *P* = .008**
	G2 glioma post-operative tumor volume ≤ 68 cm^3^ prior to adjuvant therapy	Wahl M, 2017	≤ 68 cm^3^ superior ***P* < .001**		Wahl M, 2017	≤ 68 cm^3^ superior ***P* < .001**	
	G2 glioma adjuvant chemoradiation therapy				Coburger J, 2016	–	**HR = 2.84, *P* < .01**
Adjuvant therapy NOS	G2 glioma adjuvant therapy	Gousias K, 2014	**HR = 8.115, *P* < .001**		Gousias K, 2014	**HR = 2.449, *P* = .039**	**HR = 0.105, *P* = .002**
	G2 astrocytoma adjuvant therapy following surgery at diagnosis (ref. is yes)	Jungk C, 2016	**HR = 6.25, *P* = .0010**	**HR = 7.13, *P* = .003**			
	G2 glioma adjuvant therapy and surgery at first relapse vs surgery alone				Jansen E, 2019	Adjuvant therapy and surgery superior ***P* = .0001**	
Chemotherapy	G2 glioma post-operative chemotherapy vs. no chemotherapeutic				Nitta M, 2015	**HR = 0.441, *P* = .0195**	**HR = 0.315, *P* = .0161**
					Youland RS, 2013	NS	**HR = 0.72, *P* = .008**
Radiation therapy	G2 glioma adjuvant radiotherapy (ref. no radiotherapy)	Kavouridis VK, 2020		**HR = 2.99, *P* = .001**	Kavouridis VK, 2020		**HR = 0.41, *P* < .001**
					Youland RS, 2013	NS	**HR = 0.57, *P* < .0001**
					Ius T, 2012	**HR = 0.600, *P* = .024**	
	G2 glioma immediate (ref.) vs. delayed post-operative radiotherapy				Houillier C, 2010	Delayed radiotherapy inferior ***P* < .0001**	
					Houillier C 2010	Delayed radiotherapy inferior ***P* < .0001**	
	G2 glioma post-operative radiotherapy i) Diffuse astrocytoma ii) Oligodendroglioma				Nitta M, 2015	i) NS ii) Adjuvant radiotherapy superior ***P* = .02**	
Surgical factors	G2 glioma use of intra- operative electrical stimulation with or without addition of intra-op DTI/fMRI navigation	Ius T, 2012	**HR = 0.388, *P* = .016**				
	G2 glioma use of intra- operative MRI				Kavouridis VK, 2020		**HR = 1.69, *P* = .007**
	G2 glioma surgery (ref.) vs. biopsy alone	Gousias K, 2014	**HR = 0.137, *P* < .001**		Pallud J, 2013	Surgery superior ***P* < .001**	
		Wahl M, 2017	Surgery superior ***P* = .01**		Wahl M, 2017	Surgery superior ***P* = .003**	
	G2 glioma EOR biopsy (ref.) i) STR ii) NTR iii) GTR	Goze C, 2014	i) **HR = 0.18, *P* = .031** ii) NS iii) NS	i) NS ii) **HR = 0.22, *P* = .038** iii) NS	Goze C, 2014	i) NS ii) NS iii) **HR = 0.34, *P* = .038**	i) **HR = 0.27, *P* = .021** ii) NS iii) **HR = 0.25, *P* = .025**
	G2 glioma % EOR (continuous)	Eseonu CI, 2017	**HR = 0.994, *P* = .016**	**HR = 0.979, *P* = .029**	Eseonu CI, 2017	**HR = 0.983, *P* = .005**	**HR = 0.982, *P* = .018**
		Ius T, 2012	**HR = 0.933, *P* < .0001**	**HR = 0.958, *P* = .001**	Ius T, 2012	**HR = 0.930, *P* < .0001**	**HR = 0.940, *P* < .0001**
		Majchrzak K, 2012	**HR = 0.96, *P* = .025**		Jungk C, 2016	**HR 0.23; *P* = .031**	
					Majchrzak K, 2012	**HR = 0.98, *P* = .004**	
					Scherer M, 2020	***P* < .001**	**HR = 0.98, *P* = .005**
	G2 glioma post-operative volume (cm^3^) (continuous)	Kavouridis VK, 2020	**HR = 1.02, *P* < .0001**	**HR = 1.06, *P* = .016**	Kavouridis VK, 2020		**HR = 1.01, *P* = .001**
		Scherer M, 2020	Smaller tumor volume superior ***P* = .02**		Majchrzak K, 2012	**HR = 1.01, *P* = .008**	
	G2 glioma post-operative volume (cm^3^) i) Oligodendroglioma (9 vs. ≥9) ii) IDH mutant astrocytoma (1 vs. ≥1) iii) IDH wt astrocytoma (1 vs. ≥ 1)	Kavouridis VK, 2020	Smaller tumor volume superior i) ***P* = .048** ii) ***P* = .019** iii) ***P* = .017**				
	G2 glioma % EOR i) ≥ 90% (ref) ii) 70–90% iii) <70%	Ius T, 2012	ii) **HR = 4.845, *P* = .002** iii) **HR = 19.702, *P* < .0001**		Ius T, 2012	**ii) HR = 3.402, *P* < .0001** **iii) HR = 13.60, *P* < .0001**	
	G2 glioma non-GTR (ref.) vs. GTR i) Oligodendroglioma ii) Diffuse astrocytoma IDH wt iii) Diffuse astrocytoma IDH mutant	Houillier C, 2010	NS	**HR = 0.51, *P* = .03**	Houillier C, 2010	GTR superior ***P* = .02**	
		Coburger J, 2016	***P* < .05**		Coburger J, 2016	***P* < .001**	**HR = 0.444, *P* < .001**
		Houillier C, 2010	GTR superior ***P* = .0004**	NS	Scherer M, 2020	GTR superior ***P* = .009**	
		Youland RS, 2013	GTR superior ***P* < .0001**	**HR = 0.51, *P* < .0001**	Jansen E, 2019	i) GTR superior ***P* = .002** ii) GTR superior ***P* = .037** iii) NS	
					Pal’a A, 2019	iii) ***P* = .035**	iii) **HR = 0.486, *P* = .019**
					Youland RS, 2013	***P* < .0001**	**HR = 0.44, *P* < .0001**
	G2 glioma GTR (ref.) vs. non- GTR	Jansen E, 2019	***P* = .003**	**HR 2.6, *P* = .017**	Jansen E, 2019	***P* = .001**	**HR = 1.95, *P* = .002**
	PXA GTR (ref.) vs. STR	Gallo P, 2013		**HR = 16.30, *P* = .004**	Gallo P, 2013	**HR = 4.60, *P* = .006**	**HR = 15.97, *P* = .001**
	G2 glioma first line therapy surgery vs. other	Goze C, 2014	**HR = 0.41, *P* = .042**	NS	Goze C, 2014	**HR = 0.53, *P* = .018**	**HR = 0.40, *P* = .015**
					Pallud J, 2013	**HR = 0.42, *P* < .001**	**HR = 0.44, *P* < .001**
	G2 glioma biopsy (ref.) vs. i) STR ii) GTR	Harary M, 2020	–	i) NS ii) **HR = 0.28, *P* = .02**	Gousias K, 2014	i) **HR = 0.306, *P* = .001** ii) **HR = 0.045, *P* < .001**	i) **HR = 0.234, *P* < .001** ii) **HR = 0.039, *P* < .001**
		Tom MC, 2019	i) ***P* = .002** ii) ***P* < .001**	i) **HR = 0.5, *P* = .003** ii) **HR = 0.3, *P* < .001**	Tom MC, 2019	i) NS ii) GTR superior ***P* = .002**	
	G2 glioma delta value pre-operative T2 weighted volumetric measurement compared to T1 weighted pre-operative measurement (continuous)	Ius T, 2012	**HR = 1.040, *P* < .0001**		Ius T, 2012	**HR = 1.034, *P* < .0001**	**HR = 1.021, *P* = .001**
	G2 glioma delta value pre-operative T2 weighted volumetric measurement compared to T1 weighted pre-operative measurement ≥ 30 cm^3^	Ius T, 2012	**HR = 3.699, *P* < .0001**	**HR = 1.035, *P* < .0001**	Ius T, 2012	**HR = 3.427, *P* < .0001**	
	G2 glioma post-operative T2 volumetric measurement (continuous)	Ius T, 2012	**HR = 1.022, *P* < .0001**		Ius T, 2012	**HR = 1.023, *P* < .0001**	
	G2 glioma post-operative T2 volumetric measurement i) < 10 cm^3^ (ref.) ii) 10–20 cm^3^ iii) 20–30^3^ iv) > 31 cm^3^	Ius T, 2012	ii) **HR = 3.281, *P* = .009** iii) **HR = 6.500, *P* < .0001** iv) **HR = 13.980, *P* < .0001**		Ius T, 2012	ii) NS iii) **HR = 5.842, *P* < .0001** iv) **HR = 13.061, *P* < .0001**	
	G2 glioma EOR (continuous) i) Diffuse astrocytoma ii) Oligodendroglial iii) Pooled astrocytoma and oligodendroglioma	Nitta M, 2015	i) ***P* = .0096** ii) NS iii) ***P* = .0003**		Nitta M, 2015	i) ***P* = .0007** ii) NS iii) ***P* < .0001**	
	G2 glioma IDH mutant recurrent surgery vs. no surgery at recurrence	Pal’a A, 2019	Recurrent surgery superior ***P* = .012**	NS			

Several studies did not specify the adjuvant therapy regimen used, though showed chemoradiotherapy was associated with an unfavorable effect on OS following multivariable analysis.^28,37^ Gousias et al^28^ showed a negative association between adjuvant therapy and OS, but did not conduct multivariable analyses for this outcome; only 5% of their cohort underwent either chemotherapy and or radiotherapy. In their multivariable analyses conducted for EFS however, including eloquent location as a covariate, adjuvant therapy had a favorable impact on EFS.

Conflicting results related to the role of adjuvant chemotherapy were observed; one group showed a positive association with both adjuvant chemotherapy and radiotherapy with increased EFS in multivariable analysis that included covariates age, histology, presenting symptoms, size and extent of resection.^49^ Another study showed increased EFS but no significant change in OS with adjuvant chemotherapy following LGG resection after multivariable analysis with covariates age, tumor diameter, pathology and adjuvant therapy.^41^

Few studies analyzed the role of adjuvant radiotherapy alone upon OS, though one included study demonstrated a significant negative impact on OS after multivariable analysis including age at diagnosis, molecular class, eloquent location, and post-operative residual volume.^38^ Adjuvant radiotherapy significantly improved EFS in two studies,^38,49^ and the effect was suggested to be greater with immediate as opposed to delayed radiotherapy following univariate analysis alone in two other reports.^32,33^

Non-significant prognostic variables are shown in Supplementary Table 1. Following multivariable analysis, several studies found a non-significant association between OS for LGG and adjuvant chemotherapy,^23,28,38,41,49,52,58^ adjuvant radiotherapy^22,39,41,49,52^ and combined adjuvant chemoradiotherapy.^54^

Several studies looked at the impact of surgery-related factors. Increased extent of resection compared to biopsy alone was associated with both OS and EFS in multivariable adjusted models.^29,30,46^ Extent of resection measured as either a continuous variable^27,34,45^ or lower magnitude of post-operative volumetric tumor residual^34,38^ correlated with prolonged OS and/or EFS. Several studies showed in adjusted multivariable analysis that GTR resulted in superior OS or EFS benefit compared to other resection categories,^26,32,36,49,53^ though one study showed negative effect on EFS in IDH mutant astrocytoma.^43^ One study found that first line surgical therapy compared to observation did not significantly influence OS though it favorably impacted EFS.^29^ Factors associated with positive impact on OS following univariate analysis (in absence of adjusted multivariable analysis) included: decreasing post-operative T2-weighted MRI signal volume,^34^ greater extent of resection across histological types,^26,28,33,34,37,39,41,44,48^ and smaller post-operative tumor volume.^38,39,45^

#### Grade 3 and 4 glioma

—Groupings of Grade 3 and 4 glioma in included studies may not have reflected current classification schemes that include IDH mutational status. In addition, Grade 3 glioma may or may not be included in the definition of high-grade glioma. However, grouping Grade 3 and 4 glioma best reflected the categorization used by the papers identified in this systematic review.

Table 4 summarizes disease and treatment-related factors influencing EFS and OS in HGG. Among high-grade spinal cord glioma, there was no significant influence on localized vs. regional or invasive location on OS.^56^ Oligodendroglioma histology showed superior influence on OS compared to astrocytic histology in pooled grade 2 and 3 cases following univariate analysis (no multivariable analysis reported).^57^ Grade 3 and 4 spinal cord glioma were negative influences on OS when compared to grade 2 histology.^52^ 1p19q co-deletion, IDH mutant status, low nestin level, and mitotic index less than 4% all positively impacted OS in combined grade 2 and 3 glioma cases.^54,57,58^ No EFS analysis was conducted using these variables.

Some studies included in this review showed adjuvant radiotherapy demonstrated favorable impact on OS in pooled grade 2 and 3 glioma,^58^ pooled grade 3 and 4 spinal cord glioma,^56^ and peri-ventricular HGG.^59^ STR or biopsy-only resulted in worse OS than GTR or near-total resection (NTR) in two studies.^40,54^ Though in peri-ventricular HGG STR and GTR were favorably associated with OS in univariate analysis compared to no surgery, they lost significance following adjusted multivariable analysis. Adjuvant chemoradiation positively impacted EFS in grade 2 and 3 glioma, though chemotherapy alone was not significant.^57^ Grade 2 and 3 adjuvant radiotherapy also favorably influenced EFS.^57^ One combined cohort of grade 2 and 3 glioma showed a non-significant influence of adjuvant chemoradiotherapy on OS following multivariable analysis.^54^

Excluding spinal pilocytic astrocytoma, Fakrehddine et al^52^ showed adjuvant chemotherapy significantly improved EFS in infiltrative spinal cord glioma (grades 2, 3 and 4) after adjusting for treatment modality, age at diagnosis, grade, number of spinal levels, neurological deficits and symptom duration. In the same analysis, adjuvant radiotherapy did not significantly impact EFS nor did either chemotherapy or radiation contribute to OS benefit after multivariable analysis.^52^

### Quality Assessment

Given the absence of methodological limitation reporting across studies, the QUIPS assessment tool was utilized the provide a standardized risk of bias assessment (Supplementary Table 2). Most studies (35/40) had at least 1 domain that scored in the high risk of bias category. Among included studies only 1 was prospective.^39^ Common domains for high risk of bias include study participation and adjustment for other prognostic factors.

## Discussion

This systematic review identified 40 studies that reported on demographic, disease and treatment predictors of EFS and OS among AYA glioma patients in high income countries. Despite stringent definitions utilized to capture an adequately sized AYA cohort, several included studies captured a proportion of older adults (Figure 1). This points to a severe limitation in the existing AYA glioma literature, with all interpretation limited by the potential impact of older adult glioma biology in these cohorts. In contrast, only two studies included pediatric patients.^52,53^ Furthermore, many papers scored in the high-risk bias category in at least one domain. Despite this, several patient epidemiological, disease and treatment factors with prognostic impact on EFS and OS were identified.

### Prognostication

There are important differences in glioma prognostication in adult and pediatric populations. In a national pediatric cohort study, lower tumor grade, GTR, non-brainstem location and age >1 year at diagnosis were all associated with longer OS.^60^ Recent clinical and molecular characterization has underscored the importance of single-nucleotide variant (SNV) and rearrangements in the pathobiology of pediatric LGG with SNV-driven tumors exhibiting inferior OS.^5^ Several molecular factors have important prognostic implications in pediatric LGG including mutations in BRAF V600E, *KIAA1549-BRAF* and NF-1 along with other less commonly encountered oncogenes. Identification of H3 K27M mutation in pediatric glioma portends a worse prognosis regardless of histologic diagnosis and modifies this clinical entity to WHO grade 4.^61,62^ Pathological and molecular favorable prognostic characteristics in adult glioma include IDH mutant, MGMT promoter methylation, non-astrocytoma histology or 1p/19q co-deletion and lower glioma grade when compared to IDH-WT glioma in older adults.^63,64^ Importantly, the influence of IDH mutation status in the AYA LGG is still not clear as this mutation does not portend the same prognostic importance in pediatric populations where it is encountered more rarely.^5^ Despite being highlighted as an important prognostic factor in this review, we are cognizant that this may reflect bias from inclusion of older adults, where IDH mutation is a known favorable molecular prognosticator (Figure 1). The role of IDH mutations in AYA, particulary younger AYA, remains uncertain.

Despite the AYA glioma demographic straddling the late pediatric and early adulthood age ranges, no studies in this systematic review comprehensively examined molecular prognostic markers. It is thus impossible to outline the specific prognostic impact of various molecular alterations in the AYA demographic. Instead, the literature could only confirm more the favorable impact of traditional adult prognosticators such as younger age at diagnosis, higher functional status, IDH mutant status (with limitations discussed above), lower glioma grade and 1p/19q co-deletion/ oligodendroglioma histology with limited information on clinical behavior of tumors with other molecular alterations. The effect of traditional functional status indicators such as KPS may reflect the older adults included in the review cohort. Furthermore, we have utilized previously described age parameters (15–39) for definition of AYA glioma patients; this is an assumption that will require future validation in this disease entity.^9,10^ Despite the widely accepted AYA age range, patients at the upper and lower end of the spectrum may be clinically distinct. Comprehensive molecular analyses among AYA cohort and their prognostic impact is a significant priority for future research.

### Treatment

Several surgical factors were identified as important treatment-related factors for OS and EFS among AYA glioma patients. Extent of surgical resection was identified as an important positive factor associated with EFS and OS.^26,27,29,30,32,34,36,38,40,45,46,49,51,53,54^ The degree of resection and extent-of-resection categories within each study were not standardized nor was the definition of NTR and STR across studies. However, this favorable survival influence was present in several studies after multivariable analysis when GTR or NTR was compared to other resection categories in LGG or HGG cases.^29,30,32,36,40,46,49,51,53,54^ Furthermore, the impact of surgery was demonstrated in different anatomic compartments such as spinal cord glioma,^51^ in the setting of recurrent transformed LGG^40^ and different intracranial LGG pathological subtypes,^32,49,53^ though not in peri-ventricular HGG.^59^ This is in keeping with traditional surgical principles in glioma management across the age spectrum.

The role of adjuvant therapy and its influence on OS remains unclear in the current literature. One significant limitation is heterogeneous chemotherapy regimens in tumors with differing duration, agents and timing. Indeed, some studies did not provide any details of the regimen used. Radiotherapy doses ranged between 54 and 60 Gy. Secondly, despite attempts at adjustment for confounders through multivariable analyses, many studies could not fully account for patient, disease, surgical, or institutional factors that may influence the choice of chemotherapy and radiotherapy. For example, in several LGG studies, adjuvant radiotherapy conferred a negative survival benefit.^20,23,38,43,51^ The reasons for this disadvantage may include confounders such as residual tumor and radiographic or symptomatic progression or irradiation associated complications including secondary malignancies, transformation or vasculopathies.

Discussion about the role of chemotherapy and radiotherapy in AYA glioma raises several important points. First, AYA glioma patients have historically been under-represented in clinical trials that have established current chemotherapy and radiotherapy regimens.^65–67^ Our review shows that the current literature does not guide clinicians treating AYA with LGG on whether pediatric or adult approaches are more suitable, or indeed whether a tailored approach unique to AYA is required. In both groups, treatment approaches are informed by histopathological and molecular characteristics. Many pediatric patients treated with surgery alone despite post-surgical residual disease in an effort to avoid the long-term impacts of radiation or chemotherapy.^5^ In contrast, in older adults LGG or those with residual tumor following resection, combination chemotherapy and radiation therapy is usually considered.^68^ A major challenge is the lack of studies in this review including details about the presence of pediatric-type alterations in AYA glioma,^69–71^ thus limiting any meaningful molecularly informed conclusions about adjuvant chemoradiotherapy. Whether there is a role for adjuvant therapy among AYA with LGG either totally resected or with residual disease is a crucial question that should be prioritized.

Though HGG in pediatric and adult patients may share similarities in overall prognosis, there are important differences that exist between treatment regimens and biological considerations. At a molecular level, the profile of HGG is different with distinct copy number aberrations and driver mutations in pediatric HGG compared to adults.^72,73^ Furthermore, cancer predisposition syndromes are more common in pediatric populations compared to adults. The extent to which these pediatric-type alterations and predispositions exist in AYA demographics is not well known and was not clarified through this review, thus highlighting a major gap in understanding. Stupp et al showed that adults with HGG had improved OS with adjuvant temozolomide in combination with fractionated radiotherapy compared to radiotherapy alone.^74^ Radiotherapy typically begins 3–5 weeks following surgical resection and is typically administered at 50–60 Gy in 1.8–2 Gy fractions with limited evidence suggesting any added benefit at higher doses.^75,76^ For patients with MGMT methylated promoter glioblastoma, recurrent or progressive HGG, second line alkylating chemotherapeutics may be considered.^76,77^ By contrast, the benefit of adjuvant temozolomide in the treatment of pediatric HGG is debatable. This is highlighted by contrasting two prospective trials. Cohen et al. showed temozolomide administration during and after adjuvant radiotherapy in pediatric HGG did not improve outcomes.^78^ In contrast, Jakacki et al^79^ demonstrated that children with maximally resected non-metastatic HGG treated with radiotherapy and concomitant temozolomide followed by lomustine and temozolomide adjuvant chemotherapy experienced significantly improved outcomes. Despite the complexity in decision making surrounding HGG adjuvant therapy, our review highlights that AYA-specific data to guide clinicians is lacking.

Limitations stem from the predominance of retrospective studies included in this systematic review as well as the inclusion of older adults in many study cohorts. Despite intentions to identify and assess prognostic factors in AYA glioma, the inclusion of older adults skews the results and limits generalizability. However, stricter age-based inclusion criteria would have resulted in the exclusion of nearly all studies. Pediatric glioma mutational markers were rarely examined, precluding assessment of their prognostic value in AYA populations. Our review included all CNS gliomas, including spinal gliomas, though the latter may require different treatment approaches owing to differing biology anatomical considerations. Finally, the majority of studies were classified as at high risk of bias in at least one domain.

## Conclusion

Although this study reveals some traditional factors that appear prognostically important in AYA glioma, most, including tumor grade, pathological subtype and genetic mutations such as IDH1/2, need to be considered with care given bias from the inclusion of older adults in many studies. Interestingly, the role of cytoreductive surgery remains an important prognostic factor in AYA gliomas and may not change until effective adjuvant medical therapies emerge. As such, the current literature does not provide clinicians with an evidence-based approach to treating AYA with gliomas, particularly regarding the role of adjuvant chemotherapy and radiotherapy. Available evidence is heterogenous, of mixed quality, at high risk for confounding, and predominantly derived from older adult cohorts. Prospective studies of histopathological and molecularly-defined gliomas exposed to uniform treatment including both short- and long-term outcomes will allow the identification of optimal AYA-specific glioma management strategies.

## Supplementary Material

vdac168_suppl_Supplementary_Material
