# Supplementary material for: Adolescent and young adult glioma: systematic review of demographic, disease, and treatment influences on survival
Source: Neurooncol Adv. 2022 Oct 22;4(1):vdac168. doi: 10.1093/noajnl/vdac168 (PMC9721387; doi:10.1093/noajnl/vdac168)
Supplement: vdac168_suppl_Supplementary_Material [file vdac168_suppl_supplementary_material.docx]

Supplemental Tables and Figures:

**Supplemental Table 1:** Search strategy for systematic review.

| **Database [Platform]** *Searches run July 14, 2020* | **Results** |
| --- | --- |
| **MEDLINE(R) and Epub Ahead of Print, In-Process & Other Non-Indexed Citations and Daily** [OVID] 1946 to July 10, 2020 | 4739 |
| **Embase Classic+Embase [OVID]** 1947 to 2020 Week 28 | 6750 |
| **EBM Reviews - Cochrane Central Register of Controlled Trials** June 2020**, EBM Reviews - Cochrane Database of Systematic Reviews [OVID]** 2005 to July 10, 2020 | 805 |
| **TOTAL** | **12,294** |

**Ovid MEDLINE(R) and Epub Ahead of Print, In-Process & Other Non-Indexed Citations and Daily**1946 to July 10, 2020
Search Strategy:

| **#** | **Searches** | **Results** |
| --- | --- | --- |
| 1 | glioma/ or astrocytoma/ or glioblastoma/ or diffuse intrinsic pontine glioma/ or ependymoma/ or glioma, subependymal/ or ganglioglioma/ or gliosarcoma/ or medulloblastoma/ or oligodendroglioma/ or optic nerve glioma/ | 83102 |
| 2 | (glial cell tumor? or glial cell tumour? or glioma? or astrocytoma? or astroglioma? or mixed oligoastrocytoma? or glioblastoma? or dipg brain tumor? or dipg brain tumour? or ependymoma? or subependymal glios#s or subependymoma? or ganglioglioma? or gliosarcoma? or medulloblastoma? or medullomyoblastoma? or oligodendroglioma? or oligodendroblastoma?).tw,kf. | 100659 |
| 3 | or/1-2 | 114148 |
| 4 | Adolescent/ | 2021986 |
| 5 | Adult/ | 4987577 |
| 6 | (adolescen* or teen* or preteen* or pre-teen* or youth? or young person? or young people or adult?).mp. | 6492841 |
| 7 | (AYA or AYAs).tw,kf. | 1495 |
| 8 | or/4-7 | 6492889 |
| 9 | prognosis.sh. or diagnosed.tw. or cohort:.mp. or predictor:.tw. or death.tw. or exp models, statistical/ | 2722383 |
| 10 | prognos*.tw,kf. | 613181 |
| 11 | or/9-10 | 2981228 |
| 12 | Survival/ | 4719 |
| 13 | Cancer Survivors/ | 3643 |
| 14 | Survival Rates/ | 172407 |
| 15 | progression-free survival/ | 3040 |
| 16 | disease-free survival/ | 73962 |
| 17 | surviv*.tw,kf. | 1145137 |
| 18 | or/12-17 | 1218269 |
| 19 | 3 and 8 and 11 and 18 | 7336 |
| 20 | Epidemiologic studies/ | 8353 |
| 21 | exp case control studies/ | 1089422 |
| 22 | exp cohort studies/ | 2009171 |
| 23 | Case control.tw. | 125503 |
| 24 | (cohort adj (study or studies)).tw. | 206754 |
| 25 | Cohort analy$.tw. | 8065 |
| 26 | (Follow up adj (study or studies)).tw. | 49263 |
| 27 | (observational adj (study or studies)).tw. | 107165 |
| 28 | Longitudinal.tw. | 245309 |
| 29 | Retrospective.tw. | 531943 |
| 30 | Cross sectional.tw. | 353615 |
| 31 | Cross-sectional studies/ | 331674 |
| 32 | or/20-31 | 3015479 |
| 33 | randomized controlled trial.pt. | 509322 |
| 34 | clinical trial.pt. | 523678 |
| 35 | randomi?ed.ti,ab. | 625922 |
| 36 | placebo.ti,ab. | 214930 |
| 37 | dt.fs. | 2218451 |
| 38 | randomly.ti,ab. | 337489 |
| 39 | trial.ti,ab. | 597685 |
| 40 | groups.ti,ab. | 2092772 |
| 41 | or/33-40 | 4930077 |
| 42 | animals/ | 6633540 |
| 43 | humans/ | 18579978 |
| 44 | 42 not (42 and 43) | 4682717 |
| 45 | 41 not 44 | 4297212 |
| 46 | 32 or 45 | 6348556 |
| 47 | 3 and 8 and 11 and 18 and 46 | 4918 |
| 48 | limit 47 to english language | 4739 |

**Embase Classic+Embase**1947 to 2020 Week 28
Search Strategy:

| **#** | **Searches** | **Results** |
| --- | --- | --- |
| 1 | glioma/ or astrocytoma/ or ependymoma/ or experimental glioma/ or gliosarcoma/ or oligodendroglioma/ or optic nerve glioma/ or pontine glioma/ | 88694 |
| 2 | (glial cell tumor? or glial cell tumour? or glioma? or astrocytoma? or astroglioma? or mixed oligoastrocytoma? or glioblastoma? or dipg brain tumor? or dipg brain tumour? or ependymoma? or subependymal glios#s or subependymoma? or ganglioglioma? or gliosarcoma? or medulloblastoma? or medullomyoblastoma? or oligodendroglioma? or oligodendroblastoma?).tw,kw. | 148123 |
| 3 | or/1-2 | 166692 |
| 4 | exp adolescent/ | 1656512 |
| 5 | adult/ or young adult/ | 7505297 |
| 6 | (adolescen* or teen* or preteen* or pre-teen* or youth? or young person? or young people or adult?).mp. | 8894463 |
| 7 | (AYA or AYAs).tw,kw. | 2914 |
| 8 | or/4-7 | 8894573 |
| 9 | follow-up.mp. or prognos*.tw. or ep.fs. | 3722454 |
| 10 | predictor*.tw,kw. | 581994 |
| 11 | or/9-10 | 4088751 |
| 12 | exp survival/ | 1147548 |
| 13 | cancer survivor/ | 23953 |
| 14 | survival rate/ | 251676 |
| 15 | exp progression free survival/ | 109638 |
| 16 | exp disease free survival/ | 83594 |
| 17 | surviv*.tw,kw. | 1711643 |
| 18 | or/12-17 | 1949666 |
| 19 | 3 and 8 and 11 and 18 | 10879 |
| 20 | Clinical study/ | 169808 |
| 21 | Case control study/ | 157504 |
| 22 | Family study/ | 27128 |
| 23 | Longitudinal study/ | 142049 |
| 24 | Retrospective study/ | 937748 |
| 25 | Prospective study/ | 612766 |
| 26 | Randomized controlled trials/ | 181708 |
| 27 | 25 not 26 | 606274 |
| 28 | Cohort analysis/ | 592509 |
| 29 | (Cohort adj (study or studies)).mp. | 307437 |
| 30 | (Case control adj (study or studies)).tw. | 134017 |
| 31 | (follow up adj (study or studies)).tw. | 68678 |
| 32 | (observational adj (study or studies)).tw. | 167638 |
| 33 | (epidemiologic$ adj (study or studies)).tw. | 110027 |
| 34 | (cross sectional adj (study or studies)).tw. | 218937 |
| 35 | 20 or 21 or 22 or 23 or 24 or 27 or 28 or 29 or 30 or 31 or 32 or 33 or 34 | 2757598 |
| 36 | exp clinical trial/ | 1521696 |
| 37 | randomi?ed.ti,ab. | 897534 |
| 38 | placebo.ti,ab. | 312506 |
| 39 | dt.fs. | 3902229 |
| 40 | randomly.ti,ab. | 448610 |
| 41 | trial.ti,ab. | 875548 |
| 42 | groups.ti,ab. | 2993543 |
| 43 | or/36-42 | 7786108 |
| 44 | animal/ | 1959682 |
| 45 | human/ | 22240127 |
| 46 | 44 not (44 and 45) | 1487946 |
| 47 | 43 not 46 | 7602087 |
| 48 | 35 or 47 | 9292579 |
| 49 | 3 and 8 and 11 and 18 and 48 | 6931 |
| 50 | limit 49 to english language | 6750 |

**EBM Reviews - Cochrane Central Register of Controlled Trials**June 2020**, EBM Reviews - Cochrane Database of Systematic Reviews**2005 to July 10, 2020
Search Strategy:

| **#** | **Searches** | **Results** |
| --- | --- | --- |
| 1 | glioma/ or astrocytoma/ or glioblastoma/ or optic nerve glioma/ or ependymoma/ or glioma, subependymal/ or ganglioglioma/ or gliosarcoma/ or medulloblastoma/ or oligodendroglioma/ | 1162 |
| 2 | (glial cell tumor? or glial cell tumour? or glioma? or astrocytoma? or astroglioma? or mixed oligoastrocytoma? or glioblastoma? or dipg brain tumor? or dipg brain tumour? or ependymoma? or subependymal glios#s or subependymoma? or ganglioglioma? or gliosarcoma? or medulloblastoma? or medullomyoblastoma? or oligodendroglioma? or oligodendroblastoma?).tw,kw. | 3981 |
| 3 | or/1-2 | 4058 |
| 4 | adolescent/ or adult/ or young adult/ | 357451 |
| 5 | (adolescen* or teen* or preteen* or pre-teen* or youth? or young person? or young people or adult?).mp. | 721061 |
| 6 | (AYA or AYAs).tw,kw. | 190 |
| 7 | or/4-6 | 721067 |
| 8 | prognosis.sh. | 13957 |
| 9 | diagnosed.tw. | 61047 |
| 10 | cohort.mp. | 62539 |
| 11 | predictor*.tw. | 34627 |
| 12 | death.tw. | 61576 |
| 13 | exp models, statistical/ | 15736 |
| 14 | prognos*.tw,kw. | 40036 |
| 15 | or/8-14 | 234600 |
| 16 | survival/ | 133 |
| 17 | survival rate/ | 10129 |
| 18 | disease-free survival/ | 6810 |
| 19 | surviv*.tw,kw. | 116414 |
| 20 | or/16-19 | 120965 |
| 21 | 3 and 7 and 15 and 20 | 902 |
| 22 | limit 21 to english language [Limit not valid in CDSR; records were retained] | 805 |

**Supplemental Figure 1. PRISMA diagrammatic workflow detailing process of manuscript identification, screening, eligibility and final inclusion.**

**
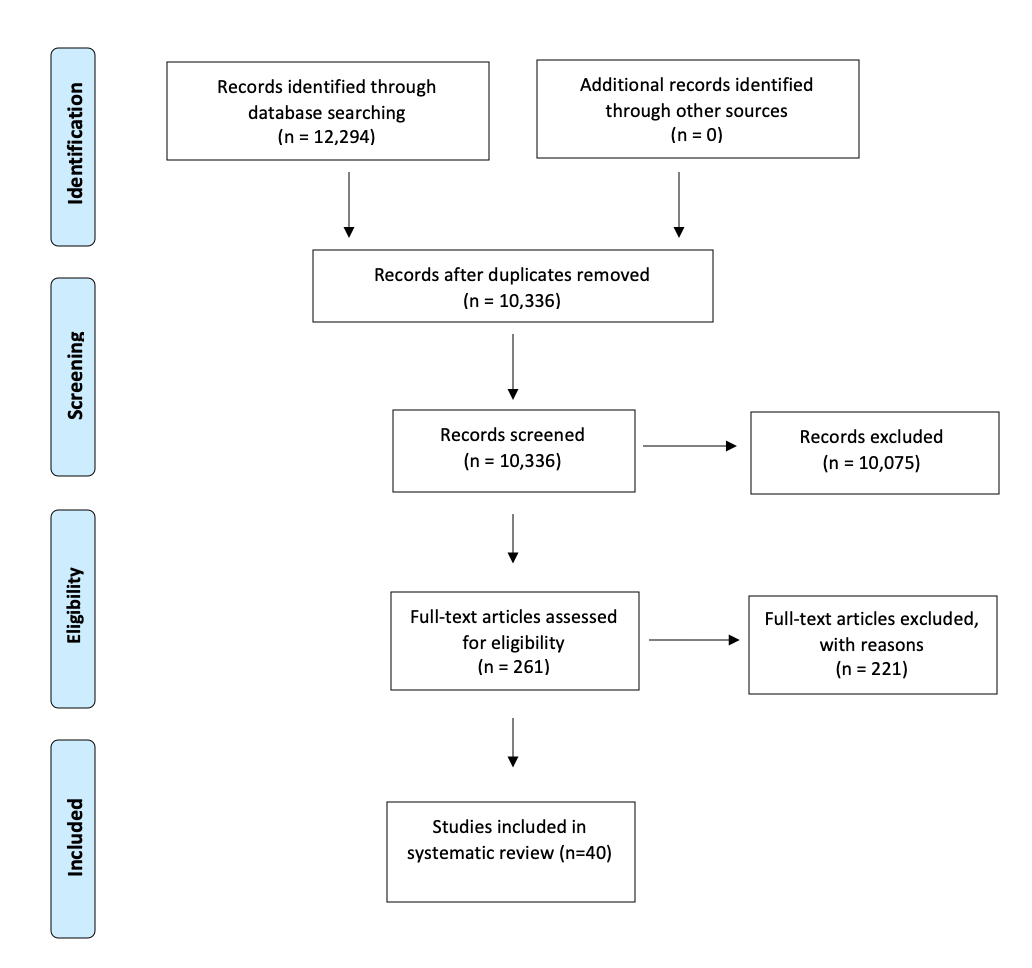
**

Reasons for exclusion:

- Outside age range
- < 20 AYA patients
- Inadequate reporting of primary outcome measure
- LMIC studies
- Case series, editorials

**Supplemental Table 2: Results of risk of bias assessment using QUIPS tool.**

|  | 1. Study participation | 2. Study attrition | 3. Prognostic factor measurement | 4. Outcome measurement | 5. Adjustment for other prognostic factors | 6. Statistical analysis and  reporting |
| --- | --- | --- | --- | --- | --- | --- |
| Ahmadi R, 2012 | High | Moderate | High | Moderate | High | High |
| Bagley JH, 2013 | Moderate | Moderate | Low | Moderate | High | High |
| Byun J, 2018 | High | Moderate | Moderate | Moderate | High | High |
| Chang EF, 2011 | Moderate | Low | Moderate | Low | Moderate | Moderate |
| Coburger J, 2016 | Moderate | Moderate | Low | Low | High | Moderate |
| Diaz-Aguilar D, 2019 | Moderate | Moderate | High | Moderate | High | High |
| Eseonu CI, 2017 | High | Moderate | Moderate | Moderate | Moderate | High |
| Fakhreddine MH, 2013 | High | Moderate | High | Moderate | Moderate | High |
| Gallo P, 2013 | High | Moderate | High | Moderate | High | High |
| Gousias K, 2014 | High | Moderate | Low | Low | Moderate | Moderate |
| Goze C, 2014 | Moderate | High | High | Moderate | Moderate | High |
| Harary M, 2020 | Moderate | Moderate | Moderate | Moderate | Low | Moderate |
| Hartmann C, 2011 | High | High | Low | Moderate | High | High |
| Hatanpaa KJ, 2014 | High | Moderate | Low | Moderate | High | High |
| Houillier C, 2010 | Moderate | Moderate | Low | Moderate | Moderate | Moderate |
| Houillier C, 2010 | Moderate | Moderate | Low | Moderate | Moderate | Moderate |
| Ius T, 2012 | High | Moderate | Moderate | Moderate | Low | Moderate |
| Jairam V, 2019 | Moderate | High | Low | Low | High | Moderate |
| Jansen E, 2019 | Moderate | Low | Moderate | Moderate | High | High |
| Jungk C, 2016 | High | Moderate | Moderate | Moderate | High | High |
| Kavouridis VK, 2020 | High | Moderate | Low | Low | Moderate | Moderate |
| Lee KJ, 2018 | Moderate | High | Moderate | Moderate | Moderate | High |
| Leibetseder A, 2013 | High | Low | Moderate | Moderate | High | High |
| Liu J, 2018 | Moderate | Moderate | Moderate | Moderate | High | High |
| Majchrzak K, 2012 | Moderate | Moderate | Moderate | Low | High | High |
| Miller JJ, 2019 | High | Moderate | Moderate | Low | High | Moderate |
| Narang AK, 2017 | High | Moderate | Moderate | Moderate | High | High |
| Nelson AJ, 2019 | High | High | Moderate | Low | High | High |
| Nitta M, 2015 | High | High | High | Moderate | High | High |
| Okita Y, 2012 | High | Moderate | Low | Moderate | Moderate | High |
| Olar A, 2015 | High | Moderate | Low | Low | High | Moderate |
| Pal’a A, 2019 | High | Moderate | Moderate | Moderate | High | Moderate |
| Pallud J, 2013 | Moderate | Moderate | Low | Low | High | Low |
| Rønning PA, 2016 | Moderate | Moderate | Moderate | High | High | Moderate |
| Scherer M, 2020 | Moderate | High | Moderate | Low | High | Moderate |
| Tom MC, 2019 | Moderate | Moderate | Moderate | Moderate | High | High |
| Tom MC, 2019 | Moderate | Moderate | Moderate | Moderate | High | Moderate |
| Wahl M, 2017 | Moderate | Moderate | Moderate | Low | High | Moderate |
| Yang W, 2018 | Low | Moderate | Low | Low | Moderate | Low |
| Youland RS, 2013 | Moderate | High | Moderate | Moderate | High | Moderate |

High/Moderate/Low: Indicates High/Moderate/Low risk of bias

**Supplement 3: List of tumors included in this review**

**Diffuse astrocytic and oligodendroglial tumors**

WHO grade 2

- [diffuse astrocytoma](https://radiopaedia.org/articles/diffuse-astrocytoma?lang=us)
  - IDH-mutant - 9400/3
    - ​[gemistocytic astrocytoma](https://radiopaedia.org/articles/gemistocytic-astrocytoma?lang=us) - 9411/3
  - *IDH-wildtype*- 9400/3
  - NOS - 9400/3
- [oligoastrocytoma](https://radiopaedia.org/articles/oligoastrocytoma?lang=us) NOS - 9382/3
- [oligodendroglioma](https://radiopaedia.org/articles/oligodendroglioma?lang=us)  - 9450/3
  - IDH-mutant, 1p19q co-deleted
  - [oligodendroglioma NOS](https://radiopaedia.org/articles/oligodendroglioma-nos?lang=us)
- WHO grade 3
  - [anaplastic astrocytoma](https://radiopaedia.org/articles/anaplastic-astrocytoma?lang=us) - 9401/3
    - IDH-mutant
    - ​*IDH-wildtype*
    - NOS
  - [anaplastic oligoastrocytoma](https://radiopaedia.org/articles/missing?article%5Btitle%5D=anaplastic-oligoastrocytoma&lang=us) NOS - 9382/3
  - [anaplastic oligodendroglioma](https://radiopaedia.org/articles/anaplastic-oligodendroglioma?lang=us) - 9451/3
    - IDH-mutant, 1p19q co-deleted
    - [anaplastic oligodendroglioma NOS](https://radiopaedia.org/articles/anaplastic-oligodendroglioma-nos?lang=us)
- WHO grade 4
  - [glioblastoma](https://radiopaedia.org/articles/glioblastoma?lang=us)
    - IDH wildtype - 9440/3
      - [giant cell glioblastoma](https://radiopaedia.org/articles/giant-cell-glioblastoma?lang=us) - 9441/3
      - [gliosarcoma](https://radiopaedia.org/articles/gliosarcoma?lang=us) 9442/3
      - [*epithelioid glioblastoma*](https://radiopaedia.org/articles/epithelioid-glioblastoma?lang=us) - 9440/3
    - IDH mutant - 9440/3 *
    - NOS - 9440/3
- [diffuse midline glioma, H3K27M-mutant](https://radiopaedia.org/articles/diffuse-midline-glioma-h3-k27mmutant?lang=us) *

**Other astrocytic tumors**

- WHO grade 1
  - [pilocytic astrocytoma](https://radiopaedia.org/articles/pilocytic-astrocytoma?lang=us) 9421/11 - WHO grade 1
  - [subependymal giant cell astrocytoma](https://radiopaedia.org/articles/subependymal-giant-cell-astrocytoma?lang=us) 9384/1 - WHO grade 1
- WHO grade 2
  - [pilomyxoid astrocytoma](https://radiopaedia.org/articles/pilomyxoid-astrocytoma?lang=us) 9425/3
  - [pleomorphic xanthoastrocytoma](https://radiopaedia.org/articles/pleomorphic-xanthoastrocytoma?lang=us) 9424/3 - WHO grade 2
- WHO grade 3
  - [anaplastic pleomorphic xanthoastrocytoma](https://radiopaedia.org/articles/anaplastic-pleomorphic-xanthoastrocytoma?lang=us) 9424/3 - WHO grade 3
- **NOS**: not otherwise specified
- **four digit code:** is from the International Classification of Disease for Oncology (ICD-O)
- **/**: the number after the slash (/) refers to biological behavior, not WHO Grade
- *****: refers to a 'new' tumor in the classification
- ***italics****:* refers to a provisional inclusion
